# Development and validation of cost-effective one-step multiplex RT-PCR assay for detecting the SARS-CoV-2 infection using SYBR Green melting curve analysis

**DOI:** 10.1038/s41598-022-10413-7

**Published:** 2022-04-20

**Authors:** Shovon Lal Sarkar, A. S. M. Rubayet Ul Alam, Prosanto Kumar Das, Md. Hasan Ali Pramanik, Hassan M. Al-Emran, Iqbal Kabir Jahid, M. Anwar Hossain

**Affiliations:** 1 Department of Microbiology, Jashore University of Science and Technology, Jashore, 7408 Bangladesh; 2 Department of Biomedical Engineering, Jashore University of Science and Technology, Jashore, 7408 Bangladesh; 3 Jashore University of Science and Technology, Jashore, 7408 Bangladesh

**Keywords:** Biological techniques, Molecular biology

## Abstract

TaqMan probe-based commercial real-time (RT) PCR kits are expensive but most frequently used in COVID-19 diagnosis. The unprecedented scale of SARS-CoV-2 infections needs to meet the challenge of testing more persons at a reasonable cost. This study developed a simple and cost-effective alternative diagnostic method based on melting curve analysis of SYBR green multiplex assay targeting two virus-specific genes along with a host-specific internal control. A total of 180 randomly selected samples portioning into two subsets based on crude and high-quality RNA extraction were used to compare this assay with a nationwide available commercial kit (Sansure Biotech Inc., (Hunan, China)), so that we could analyze the variation and validity of this in-house developed method. Our customized-designed primers can specifically detect the viral RNA likewise Sansure. We separately optimized SYBR Green RT-PCR reaction of N, E, S, and RdRp genes based on singleplex melting curve analysis at the initial stage. After several rounds of optimization on multiplex assays of different primer combinations, the optimized method finally targeted N and E genes of the SARS-CoV-2 virus, together with the β-actin gene of the host as an internal control. Comparing with the Sansure commercial kit, our proposed assay provided up to 97% specificity and 93% sensitivity. The cost of each sample processing ranged between ~2 and ~6 USD depending on the purification level of extracted RNA template. Overall, this one-step and one-tube method can revolutionize the COVID-19 diagnosis in low-income countries.

## Introduction

The COVID-19 outbreak originated in China in late 2019 quickly turned into a global pandemic by early 2020^1^. The etiological agent, severe acute respiratory syndrome coronavirus 2 (SARS-CoV-2), spreading at faster-than-ever speed now demands a low-cost but accurate diagnosis to stop person-to-person transmission. Commercial or in-house RT-PCR based on TaqMan chemistry, the gold standard for detecting SARS-CoV-2 for its higher specificity and correlation of the viral load with the cycle threshold value, is the only reliable way for detecting SARS-CoV-2. However, the major pitfall is its high cost because of dual-labeled fluorescent probes^2–4^. Thus, as a low-cost alternative, SYBR Green (a non-specific, cheap, and dsDNA-binding intercalating dye) based RT-PCR method can be used. SYBR green method has some issues of non-specific signaling by binding primers to the unwanted regions of the template, formation of primer-dimer, and presence of remaining segmented templates. The non-specificity of this method can be overcome by amplicon-specific melting-curve analysis. Other pre-requisites needed to improve the performance and quality of the SYBR green method comparable to the TaqMan assay are high-performance primer sets, modified user design, and experimental optimization^5^.

Several research studies have developed the SYBR Green-based detection method as a cheaper substitute to the TaqMan-based approach for detecting SARS-CoV-2^5–13^. Notably, none has included the multiplex reaction's internal control for host-specific amplification. We introduced the host-specific (internal control) primer sets and virus-specific ones to mimic standard TaqMan assays for COVID-19 diagnosis.

The variations in the RNA extraction method influence the result of virus detection in SYBR green assay^14^. The high-performance RNA extraction method facilitates the confirmation of virus-specific genes, whereas quick RNA extraction by a release buffer raises the possibility of false-negative results. Nevertheless, different worldwide accepted commercial RT-PCR kits suggest the prior quick RNA extraction method to diagnose the maximum number of patients per day. The high-efficiency RNA extraction method needs more time and more complicated steps. Previous SYBR Green-based studies used a high-performance RNA extraction system and converted viral RNA into cDNA for the final RT-PCR^5–8,12^. Considering this issue, we compared both types of extraction methods in this study (crude and column-based) and evaluated the efficiency of our in-house SYBR green assay.

This study aims to develop and validate an easy and inexpensive SYBR Green-based method focusing on melting-curve to detect SARS-CoV-2 specifically. Initially, we used four in-house designed primer sets in different combinations against N (nucleocapsid), E (envelope), RdRp (RNA dependent RNA polymerase), and S (spike glycoprotein) genes, including the internal control specific to the host (GAPDH: Glyceraldehyde 3-phosphate dehydrogenase and β-actin: beta-actin). Finally, we developed a multiplex SYBR-Green method to identify N,E and β-actin genes, interpreted through three distinct melting-curve peaks. We then compared this approach with one of the TaqMan-based one-step real-time PCR kits (Sansure Biotech Inc.), which the Government of Bangladesh provided for all COVID-19 testing laboratories.

## Results

**Figure 1:**
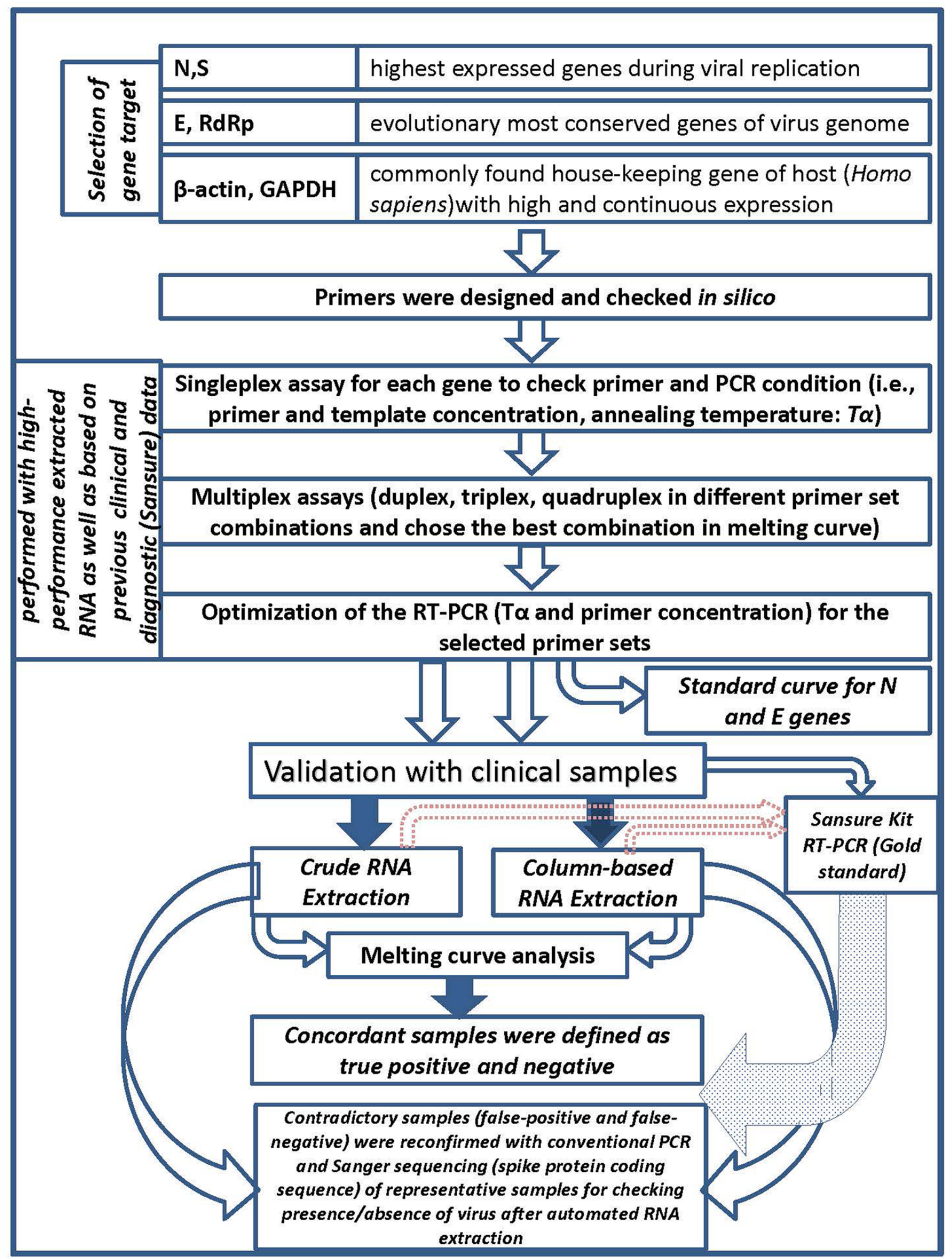
Overall workflow of the protocol starting from the primer design scheme to finally validate the assay.

In brief, the primer sets were carefully designed against specifically chosen viral genes. We initially standardized the RT-PCR condition for each gene and then targeted multiple genes for conducting multiplex PCR by combining the virus-specific primer sets along with a house-keeping gene of humans as the internal control. Optimization of the annealing temperature and concentrations of those primers and using melting curve analysis mainly sifted out the non-specificity issues of the SYBR green method. We then validated the assay with a total of 180 clinical samples where an equal number of samples were extracted either by crude or column-based RNA extraction system. To assess the effectiveness of our assay, the Sansure kit was considered as the gold standard here. The overall representation of the workflow is delineated in Fig. 1.

### Primer sequence validation

The oligo-analyzer tool showed no stable secondary structure, hairpins, homodimers, and cross-dimer formed within the primer set sequences. The result of the Primer-BLAST tool from the NCBI showed that all the genes’ primer set only matched with the expected target size of the SARS-CoV-2 virus genome (reference strain Wuhan-Hu-1), indicating that the performance of the SYBR Green assay would be specific enough for SARS-CoV-2 detection. Searching against the human genome and other pathogens showed no similarity, bolstering that the viral genome segments alone would be amplified (Supplementary Table S1). We found no mutation that engendered the new variants within the primer-binding region of the viral genome. Mutations with low frequency were identified within the last five bases of spike forward and reverse primers, while RdRp, N, and E targeted primers were void of end site mutations. The result of the searches were represented in the Supplementary Table S1.

### Development of singleplex SYBR green assay

**Figure 2:**
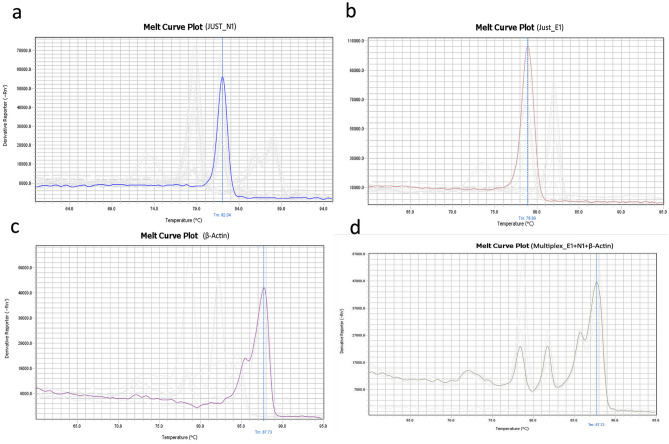
Melting curve plot of singleplex and multiplex assay. (**a**) Melting curve of N gene of COVID-19 Positive sample; (**b**) melting curve of E gene of COVID-19 Positive sample; (**c**) melting curve of Housekeeping gene β-Actin; (**d**) Melting Curve of Multiplex E + N + β-Actin genes of Covid-19 Positive Sample.

Using JUST_N1, JUST_E1, JUST_S1, JUST_RdRp1, β-actin, and GAPDH primer set individually, and singleplex assays were at first performed for four randomly selected clinical samples (three positive low-Ct and one negative, previously detected by Sansure RT-PCR kit). Specific desired band position for each gene target was observed in the electrophoresis gel as mentioned in the Table 1 (Table 1 and Supplementary Fig. S1). In the melting curve analysis for positive samples, the amplicon of JUST_N1, JUST_E1, JUST_S1, and JUST_RdRp1 primer set produced a specific melting temperature (Tm) peak at 82.32 ± 0.17 °C, 79.40 ± 0.31 °C, 76.52 ± 0.17 °C, and 77.57 ± 0.17 °C, respectively (Fig. 2 and Supplementary Fig. S2). However, a distinct dissociation curve generated for the negative samples suggested a non-specific signal or primer-dimer formation (Supplementary Table S2). The amplicon of housekeeping gene β-actin and GAPDH produced a specific Tm peak at 85.78 ± 0.24 °C and 87.59 ± 0.18 °C (Fig. 2) and 82.95 ± 0.036 °C (Supplementary Fig. S2), respectively, for both positive and negative samples. Exact details of the information were shown in Supplementary Table S2.

**Table 1:** List of the primers used in this study.

Primer name	Seq (5ʹ–3́ʹ)	Tm (°C)	Reference	Product size	Region within SARS-CoV-2 gene	Regions in SARS-CoV-2 genome
JUST_E1_F	ATTCGTTTCGGAAGAGACAGG	63	This study	117	02–29	26,254–26,274
JUST_E1_R	CGCACACAATCGAAGCGC	62.3	This study		106–123	26,350–26,367
JUST_N1_F	ACCCAATAATACTGCGTCTTGG	63.5	This study	138	135–156	28,409–28,430
JUST_N1_R	GGTAGCTCTTCGGTAGTAGCC	64.4	This study		253–273	28,527–28,547
JUST_RdRp1_F	GTACTGATGTCGTATACAGGGC	63	This study	104	79–100	13,255–13,276
JUST_RdRp1_R	CTTCGTCCTTTTCTTGGAAGCG	64.7	This study		162–183	13,338–13,359
JUST_S1_F	ACAACCAGAACTCAATTACCCC	63.5	This study	66	55–76	21,617–21,638
JUST_S1_R	TGTCAGGGTAATAAACACCACG	63.3	This study		100–121	21,663–21,684
GAPDH_F	CAATGACCCCTTCATTGACC	61.7	Tsubouchi et al. (2017)^21^	159		
GAPDH_R	TTGATTTTGGAGGGATCTCG	60.6	Tsubouchi et al. (2017)^21^			
β-actin F	CCCAAGGCCAACCGCGAGAAGAT	61.4	Law et al. (2005)^22^	219		
β-actin R	GTCCCGGCCAGCCAGGTCCAG	61.3	Law et al. (2005)^22^

### Optimization of multiplex SYBR green assay

Considering two positive clinical samples, we used JUST_N1, JUST_E1, JUST_S1, JUST_RdRp1, β-actin, and GAPDH primer sets to conduct duplex assay in nine different combinations: JUST_N1 + JUST_E1, JUST_N1 + JUST_S1, JUST_N1 + JUST_RdRp1, JUST_E1 + JUST_S1, JUST_E1 + JUST_RdRp1, JUST_S1 + JUST_RdRp1, JUST_N1 + GAPDH, JUST_E1 + GAPDH, and JUST_S1 + GAPDH; triplex assay in five different combinations: JUST_N1 + JUST_E1 + JUST_S1, JUST_N1 + JUST_RdRp1 + JUST_S1, JUST_N1 + JUST_RdRp1 + JUST_E1, JUST_N1 + JUST_E1 + β-actin, and JUST_N1 + JUST_E1 + GAPDH. The quadruplex assay was performed using primer sets combined with JUST_N1 + JUST_E1 + JUST_S1 + JUST_RdRp1 for one of those positive samples (Supplementary Table S2).

In the duplex assays, the amplicon of JUST_N1 + JUST_E1; JUST_N1 + JUST_S1; JUST_N1 + JUST_RdRp1; JUST_E1 + JUST_RdRp1; JUST_N1 + GAPDH; JUST_E1 + GAPDH, and JUST_S1 + GAPDH produced specific Tm peak at (81.58 ± 0.97 °C and 78.30 ± 0.85 °C), (81.97 ± 0.43 °C and 75.59 ± 0.27 °C), (82.09 ± 0.18 °C and 76.97 ± 0.15 °C), (78.90 °C and 76.50 °C), (82.26 °C and 83.90 °C), (78.89 °C and 82.42 °C), and (75.82 °C and 82.42 °C), respectively. But the amplicon of JUST_E1 + JUST_S1 and JUST_S1 + JUST_RdRp1 produced only a specific Tm peak at 78.95 °C and 77.01 °C, respectively, which posed that the amplicon of JUST_S1 was not properly amplified in those cases.

In the triplex assays, the amplicon of JUST_N1 + JUST_E1 + JUST_S1; JUST_N1 + JUST_RdRp1 + JUST_S1, and JUST_N1 + JUST_RdRp1 + JUST_E1 produced two specific Tm peaks at (82.42 °C and 76.02 °C), (82.27 °C and 77.23 °C), and (82.27 °C and 79.07 °C), respectively. Notably, three specific amplicons were appropriately produced in the triplex assay of JUST_N1 + JUST_E1 + β-actin and generated Tm peak at 81.82 °C, 78.49 °C, and (85.78 °C and 87.73 °C – a signature Tm peak for β-actin), respectively. In contrast, the amplicon of the housekeeping gene GAPDH combined with JUST_N1 and JUST_E1 in the triplex assay was not amplified properly and produced two specific Tm peaks at 82.20 °C and 78.43 °C, respectively. Finally, in the quadruplex assay of JUST_N1 + JUST_E1 + JUST_S1 + JUST_RdRp1, only two amplicons were generated and produced two specific Tm peaks at 82.12 °C and 77.54 °C.

**Figure 3:**
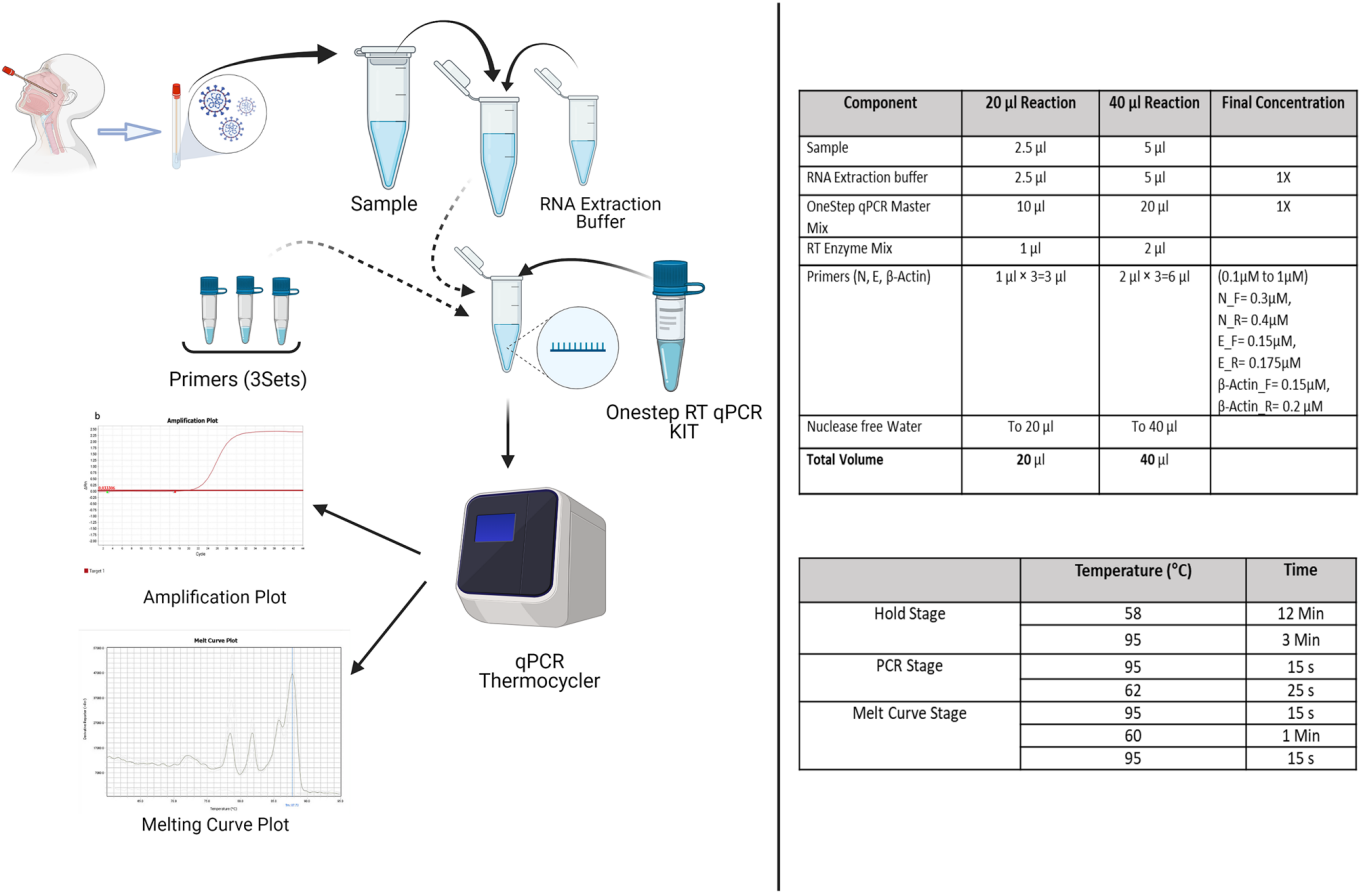
A simple schematic diagram for the standard working procedure of SYBR green method. Created with BioRender.com.

Since the amplicon size produced by using the primer set JUST_S1 is small (66 bp), it produced a Tm peak at a lower temperature. A similar Tm peak also appeared due to primer-dimer or non-specific amplified or fragmented products. We thus could not distinguish the S-gene-specific amplified product. There had also arisen some cases of mutations within the last 5 bases of the S targeting primer region (Supplementary Table S1) during the experiment. Hence, we eliminated the S targeting primers from the further multiplex study. We also omitted JUST-RdRp1 from multiplex assay for its small product size (104 bp) and less accurate result in the melting curve when run with other gene targeting and host-specific primer sets in the respective triplex assays. Housekeeping gene target GAPDH was omitted from the final multiplex assay since it produced a closer Tm peak with JUST_N1 and was not amplified efficiently with the JUST_E1 combination. Therefore, we selected JUST_N1 + JUST_E1 + β-actin primer combination for the final multiplex SYBR Green assay to detect nucleocapsid and envelope gene of SARS-CoV-2 and beta-actin gene of the host as the internal control. The result is summarized in Supplementary Table S2 and Supplementary Fig. S3. The schematic representation of the optimized protocol is shown in Fig. 3.

### Validation of in-house established multiplex SYBR Green assay with clinical samples

After confirming the primer set for multiplex reaction, we intended to optimize the method for primer concentrations and PCR conditions. For initially establishing optimal SYBR green-based protocol, we used three positive samples and one negative sample as previously confirmed by Sansure. The singleplex qRT-PCR was performed using different sets of primer (JUST_N1, JUST_E1, JUST_S1 and JUST_RdRp1) at different concentrations (50–250 nM) and different annealing temperatures (60–70°C). In our study, the optimal condition of annealing temperature was set at 62 °C and 200 nM primer concentration. We also lessened the annealing and extension time (combinedly 25 s instead of 1 min as per standard protocol) to reduce the primer dimer formation and get a higher quantity of target amplicons.

In this study, the amplicon of JUST_N1 primer and JUST_E1 primer produced a specific Tm peak at 81.8 ± 0.40 °C and 78.4 ± 0.33 °C in the melt curve for positive samples. For internal control, β-actin primer amplified product generated specific Tm peaks in two positions at around 85.8 ± 0.3 °C and 87.8 ± 0.3 °C, that is a signature peak (Fig. 2). However, a clearly distinct dissociation curve was generated for negative samples that varied with the melt curve as identified for the positive samples, suggesting non-specific signals or the formation of primer-dimers in the melt curve peaks for the negative samples. This is the crucial parameter in the analysis of the specificity of curves for the SYBR Green methodology.

### Assessment of SYBR green assay against Probe-based method (Sansure kit): crude RNA extraction method

**Figure 4:**
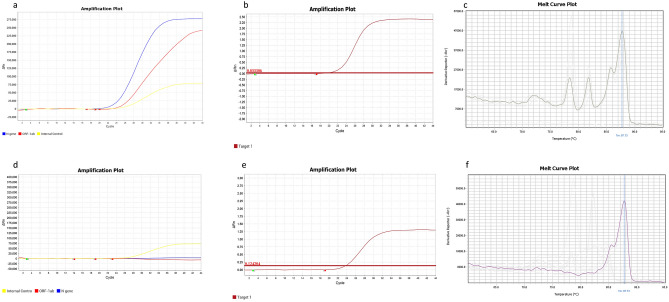
RT-PCR results for both assays. (**a**) Positive result for TaqMan Based method; (**b**) positive result for N, E and β-Actin genes in SYBR Green Based Method (linear View of fluorescence); (**c**) positive result for N, E and β-Actin genes in SYBR Green Based Method (Melt curve plot) (**d**) negative result for TaqMan Based method (only Internal control peak); (**e**) negative result for SYBR Green Based Method (linear View of fluorescence); (**f**) negative result for SYBR Green Based Method (Melt Curve Plot); Target 1 in sub-figure (**b**) and (**e**) denotes N + E + β-Actin where the reporter is SYBR. In sub-figure, (**a**) and (**d**), the blue, red and yellow color denote the N gene, ORF1ab gene and Internal control specific probe, respectively.

We assayed 90 samples from COVID-19 cases with Sansure kit and our optimized SYBR Green-based one-step RT-PCR protocol. The RNA was extracted using crude method beforehand. In both methods, 49 and 8 samples were positive and negative, respectively (Table 2 and Fig. 4). We observed rest of the 33 samples with contradictory results (27 false-positive and 6 false-negative) as shown in Table 2 and in Appendix 1 in details. To reconfirm the presence of virus, we performed the targeted PCR amplification of spike protein coding sequence for these 33 conflicting samples and did sanger sequencing for three representative samples: one from concordant positive (164.02), false-positive (164.36), and false-negative (165.104) each group. Then, we identified sequence matching with the SARS-CoV-2 spike in BLAST (> 99%) and MEGA7 based alignment (Supplementary Fig. S5 and S6).

**Table 2:** Results of samples for crude and column-based RNA extraction methods.

SYBR Green-based protocol	TaqMan based multiplex real-time PCR assay	No. of samples	Amplified for S gene
**Result of crude RNA extraction method**			
Positive	Positive	49	–
Negative	Negative	8	–
Negative	Positive	6	2
Positive	Negative	27	9 (undetermined 8^a^)
Total		90	
**Result of column-based RNA extraction method**			
Positive	Positive	61	–
Negative	Negative	25	–
Negative	Positive	2	0 (undetermined 1^a^)
Positive	Negative	2	0
Total		90	

^a^Undertermined results were designated based on spike coding sequence targeted amplification where we could not identify a particular band as positive or negative for the gene.

Among 33 samples, we designated eight of them as undetermined where the clear amplified product could not be confirmed. We found a 47% (9/19) positive and 67% (4/6) negative results for spike amplified products corroborated with our SYBR-green based assay. On the other hand, a 33% (2/6) positive and 53% (10/19) negative results accorded with the Sansure kit method (Table 2, Supplementary Fig. S5, and Appendix 1).

### Assessment of SYBR Green assay against probe-based method (Sansure kit): column-based RNA extraction method

We assayed another 90 samples of COVID-19 positive and negative cases maintaining the column-based extraction protocol. In this case, the number of concordant positive and negative samples was 61 and 25, respectively. Two samples were false-positive and false-negative in each case (Table 2 and Appendix 2). After spike gene specific amplification, we found three of them negative and one undetermined (Supplementary Fig. S5, Table 2 and Appendix 2). The triplicate runs of the multiplex assay showed consistent results for all the samples except four samples (Appendix 2).

### Spike-gene specific conventional PCR and Sanger sequencing

The electrophoresis gel showed specific clear band (228 bp) for the samples where the virus was present (Supplementary Fig. S5, Appendices 1 and 2). We designated nine samples as undetermined through this process where the gel image could not represent exact results (Table 2). The sanger results showed the similarity of the sequence to the SARS-CoV-2 (Supplementary Fig. S6).

### Comparison between crude and column-based RNA extraction approaches

The values of the sensitivity, specificity, positive likelihood ratio, negative likelihood ratio, positive predictive value, negative predictive value, and kappa index of the SYBR Green assay for both crude and column-based RNA extraction approaches were presented in Table 3. By column-based extraction, the sensitivity and the specificity of the SYBR green assay raised to 7.74% (96.83–89.09)% and 69.73% (92.59–22.86)%, respectively, when we considered Sansure solely as the gold standard. The kappa index of the crude vs column-based extraction also varied (0.134 vs 0.89). Considering the results of spike PCR amplification and further Sanger sequencing of representative samples for the false-positive and false-negative samples, the values increased (Table 3). To be more precise, the difference in specificity increased 38.31% (92.86–54.55)%. The approximate cost of detection for the crudely extracted sample was < $2, but for the column-based detection, its cost was ≤ $6 (Supplementary Table S5).

**Table 3:** Operating characteristics of in-house SYBR green assay.

Extraction method	Sensitivity	Specificity	PPV	NPV	LR +	LR-	Kappa index
Quick extract (n = 90)	89.09 (96.67^a^)	22.86 (54.55^a^)	64.47 (85.29^a^)	57.14 (85.71^a^)	1.15 (2.13^a^)	0.48(0.06^a^)	0.134 (0.579^a^)
Column based extraction (n = 90)	96.83 (100^b^)	92.59 (92.86^b^)	96.86 (96.83^b^)	92.59 (100^b^)	13.07 (14.0^b^)	0.03 (0.0^b^)	0.894 (0.947^b^)

*PPV* positive predictive value, *NPV* negative predictive value, *LR* + positive likelihood ratio, *LR − *negative likelihood ratio.

^a^Considering Sansure for condodant samples and S gene amplification for contradictory false-positive and negative samples as the standard, respectively. The sample number here would be 82 (n = 82) due to undetermined results of eight samples in spike amplification step.

^b^Considering Sansure as standard for 86 concordant samples and S gene amplification as standard for the four contradictory samples. The sample number would be 89 (n-89) here due to one undetermined result in PCR and gel run.

### Melting curve analysis

In crude extraction methods, both N and E gene-specific peaks were found in 32 out of 76 concordant samples, and either N or E gene was detected in rest of the 44 samples (Supplementary Table S4). The mean value of derivative reporter for these concordant positive samples (18,195.72 ± 5564.33) was higher by 2,283 than the false-positive (SYBR positive-Sansure kit negative) (15,912.87 ± 5085.20) on average with a similar standard deviation of ~ 5000 based on E gene peak found in 43 concordant and 16 false-positive samples. In the case of N genes in SYBR positive-Sansure kit positive samples, there were only 10 samples out of 28 where the threshold of derivative reporter went up and beyond 20,000, whereas there was only one such example out of 11 in SYBR positive-Sansure kit negative samples (Appendix 1 and Supplementary Table S4).

In column-based extraction method, we detected Tm peak for both N and E in 48 samples out of 63 positive ones (61 concordant and 2 false-positive in SYBR Green assay) and confirmed Tm peak of only N gene for 15 positive (13 concordant and 2 false-positive in SYBR Green assay) samples. The mean value of N gene specific derivative reporter for concordant positive samples was 27,280.24 with a standard deviation of 7023.48, whereas derivative reporter of both two SYBR positive-Sansure kit negative samples were below 10,000 (9223.71 ± 705.18). In concordant positive samples, the mean derivative reporter of E gene was 10,469.27 with a standard deviation of 2239.16, whereas E gene was not detected in two SYBR positive-Sansure negative samples.

### Standard curve analysis

**Figure 5:**
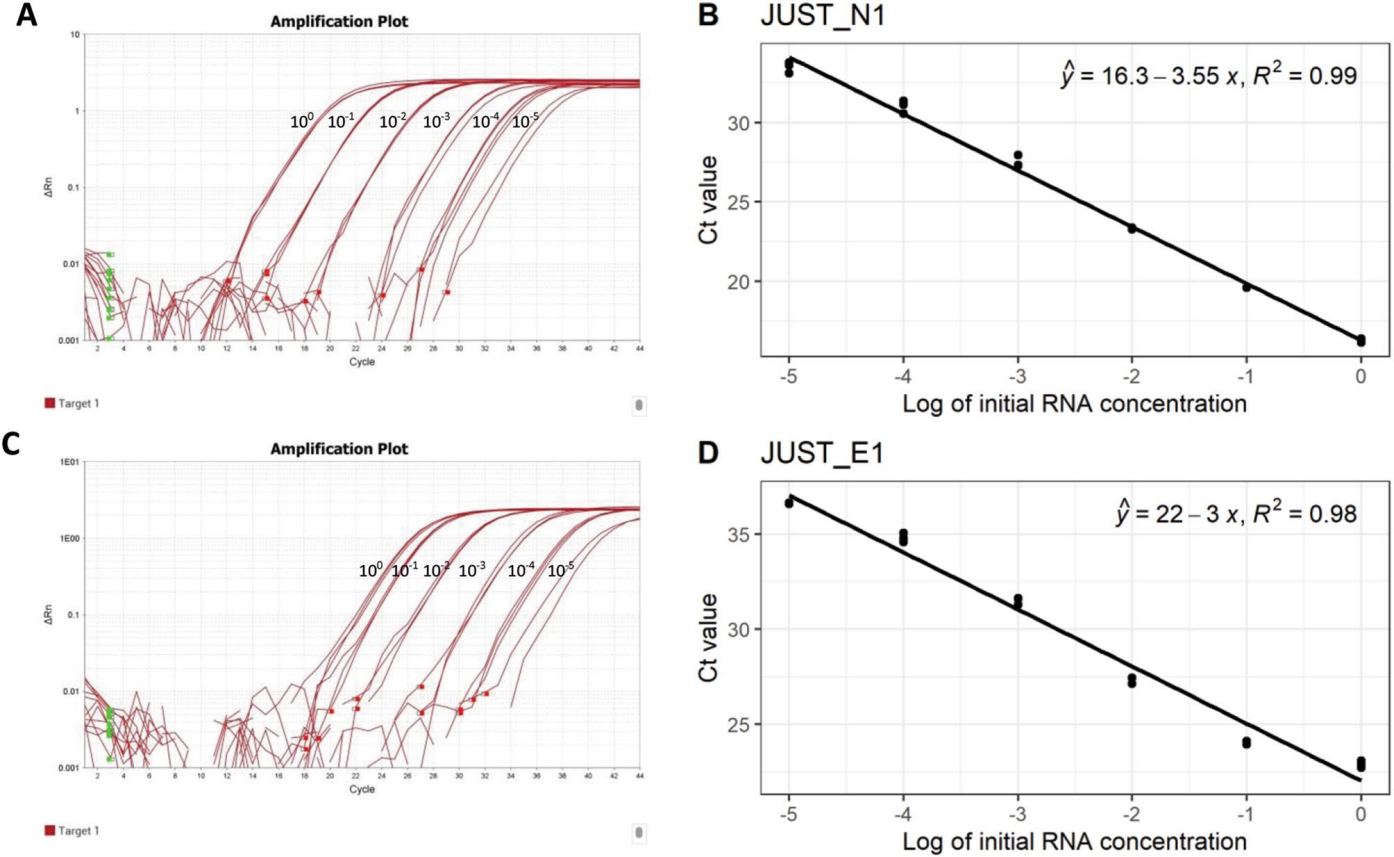
Standard Curve of JUST N1 and JUST E1 genes. (**a**) Amplification plot of JUST N1 gene (Linear View) in different dilutions (10^0^–10^–5^); (**b**) standard curve of JUST N1 gene (**c**) Amplification plot of JUST E1 gene (Linear view) in different dilutions (10^0^–10^–5^) (**d**) Standard curve of JUST E1 gene The coefficient of determination (R^y^) and linear regression curve (y) were determined.

In the standard curve, the mean Ct value of technical replicates for JUST_N1 were 16.28, 19.61, 23.32, 27.52, 31.02 and 33.48, and for JUST_E1 were 22.89, 24.06, 27.34, 31.50, 34.80 and 36.62 in 10^0^,10^–1^, 10^–2^, 10^–3^, 10^–4^, and 10^–5^ dilution of template RNA (Fig. 5). The R^2^ value of the N and E were 0.99 and 0.98, respectively, for gene-specific RT-PCR showing the curve fitting the data well. The slope value of the N gene based curve (-3.55) showed a good correlation between viral RNA numbers and Ct values (Fig. 5). For E gene, we did not get a better slope value (-3.0), given that this gene is present in low copy number in virus-infected cell. Since the N or E gene RNA transcripts within the cell other than a part of viral genome do present within the samples, we cannot correlate our data with viral copy number directly.

## Discussion

COVID-19 will continue worldwide in the foreseeable future. Apart from regular diagnostic purposes in the health sector, regular monitoring of the disease is necessary for many other sectors through valid and recognized scientific testing (i.e., real-time PCR). Even after the waning of the pandemic in the future, the COVID-19 testing will continue due to safety issues, research purposes, and the possibility of emerging vaccine-escape mutants. Developed countries can test a vast number of samples every day to control virus spread. In contrast, developing countries are still facing the obstacle of diagnosing a considerable number of patients. The testing cost is also higher in developing countries because of no raw material manufacturing industries and cross-border custom tax issues. Nevertheless, laboratories and diagnostic centers prefer the high-price probe-based method to detect SARS-CoV-2. The main reason is the absence of an alternative, low-cost, and comparably effective reverse transcription real-time PCR (RT-PCR) assay. We thus developed and validated a multiplex SYBR Green method to mimic the nationwide regularly used Taqman-based commercial kit (Sansure Biotech Ltd.) assay, even at a low-priced budget.

Notably, most previous studies were performed in two-step processes (cDNA preparation and RT-PCR reaction in different tubes). We performed those in a one-step reverse transcription RT-PCR using a single tube (cDNA preparation and PCR in the same tube). For instance, Gomez et al. (2020) and Dorlass et al. (2020) converted extracted RNA into cDNA first and then performed the PCR with the cDNA template^7,12^. This one-step approach eases the workflow, expedites overall processing, and reduces the chance of contamination.

Four customized primer sets against N, E, RdRp, and S genes of SARS-CoV-2 were employed for two reasons. The N and S are the most abundantly expressed transcripts during viral replication^15^, and the E and RdRp are the least evolving genes due to minimized effects of the selective pressure^16^ (Fig. 1). We could detect the specific genes of only SARS-CoV-2 with these primer sets, provided that other human coronaviruses might present in the clinical samples. However, we found a partial matching of the JUST_RdRp1, JUST_N1, and JUST_E1 primers with the SARS coronavirus Tor2 genome, but SARS was not detected in humans and other animals after 2005^17^. Moreover, our designed primers targeted the SARS-CoV-2 solely through screening out the mutations present among the GISAID global viral sequences within the last five bases of the 3´-end of the primer. The primers were also screened against the co-variants and clade-featured mutations that independently originated in different parts of the world, and notably, those mutants emerged after most of the previous SYBR green-based methods^18^. The finally selected primers (JUST_N1 and JUST_E1) to detect SARS-CoV-2 in the optimized multiplex reaction were also been found effective against the recently circulating and emerged variants of concern (VoC), such as Delta and Omicron (Supplementary Table S1). Thus, this assay can effectively detect the variants.

Another supremacy of our study is the multiplexing of viral N and E genes with host-specific β-actin gene. As an internal control, β-actin here was used for the same purpose as in TaqMan assays for confirming the human sample and whether the sampling has been collected accurately from the nasopharyngeal swab. This design engendered a unique approach and was not present in any existing SYBR Green-based study for COVID-19 diagnosis. However, the minor drawback of a multiplex SYBR green system is unable to predict the presence or absence of any particular gene or primer-dimer by specific Ct-value. Melting curve-based detection, wherein the dissociation points of three genes are distinct, and any flat curve on that melting point predicted the absence of the specific gene in the sample, herein has solved the issue. Hence, our study is superior to other studies in the imitation of standard TaqMan assay to separate the disparate COVID-19 positive and negative samples. Pereira-Gómez et al. (2021) used primer sets against ORF1b-nsp14 or N gene targets using melting curve analysis to differentiate primer-dimer from the target product amplification^12^. Park et al. (2020) established a multiplex cost-effective SYBR Green-based method using primer sets targeting RdRp, N, E, and S genes while suggesting melting curve analysis for confirming the specific target product amplification. However, their works did not include the internal control particular primers in the multiplex reaction, so those assays will not be appropriate for standard diagnostic purpose^10^.

The peak(s) in the melt curve shows the presence of a particular gene-targeting amplicon(s), and the derivative reporter is a measurement for the approximate quantity of that target gene(s). The melting curve analysis can also help identify mutations occurring within the target region of the amplicon^12^. A slight deviation from the average melting temperature in the dissociation curve for the N (81.8 ± 0.40 °C: crude; 81.4 ± 0.6 °C: column-based) and E (78.4 ± 0.33 °C: crude; 77.5 ± 0.6 °C: column-based) genes would mean minimal or no sequence variation among the viruses present in different clinical samples^19–21^. In the crude RNA extraction group, the difference between the derivative reporters (mean = 2283) of the concordant positive (SYBR positive-Sansure kit positive) and false-positive (SYBR positive-Sansure kit negative) samples was most probably due to a low viral concentration in the contradictory false-positive samples. Another possibility could be the presence of PCR inhibitors within samples after this quick RNA extraction. These reasonings were more evident from the melting curve results of column-based RNA extraction samples where both N and E genes were detected in 76% (48/63) of cases in contrast to only 42% (32/76) crude extracted SYBR Green positive samples. The difference of mean derivative reporters was also 18,057 (27,280–09,223) between concordant positive and false-positive cases. Even though we used a low amount of RNA for the assay (up to 7 μl), we derived better results in virus detection showing a lower value of derivative reporter for the false-positive contradictory samples (Appendix 1).

SYBR Green RT-PCR assays after crude and column-based RNA extraction exhibited an entirely different picture in terms of specificity and positive likelihood ratio (LR +). Even after considering spike amplification and Sanger, we found the specificity range up to ~ 75% at 95% CI (Supplementary Table S3). The reason is that these statistical parameters heavily rely upon the false-positive value. We found more false-positive results for our SYBR Green assay in crude extraction system-based assay. There could be two-fold reasons for this issue: false-negative results for those samples in Sansure kit and/or S-gene amplification and a non-specific product amplification in the SYBR Green assay. In a crude extraction system, the chance of getting positive results in Sansure kit-based PCR will be lower because the possibility of inhibiting the fluorescent probe in Sansure by extraction system-based inhibitors is higher. The lowest Ct value of those contradictory samples for both crude and column-based (≥ 35) was the proof (Appendices 1 and 2). It is sometimes difficult to get concrete positive results in conventional PCR where RT-PCR failed. The nine undetermined results in 31 spike gene-specific conventional PCR indicated such an issue. The undetermined results in the gel run also matched with the RT-PCR invalid result where the guaranteed amplification could not be ensured. Because of these reasons, more positive results might be found in SYBR Green RT-PCR compared to Sansure and Spike gene-based amplification.

Although the fluorescence probe-based RT-PCR is advantageous for specific target gene detection, any interfering molecule for the fluorophores and inhibitors to polymerase’s 5′-3′ exonuclease activity decreases its efficiency. The Sansure kit has such issues that can lead to underestimating viral RNA quantity or even false-negative results^22^ even with high-performance extracted RNA in some cases^23^ The low amount of viral RNA and extraction-related fluorescence-inhibitors made it more challenging to identify the viruses in Sansure assay than SYBR Green or conventional PCR.

The RT-PCR has incredibly advanced molecular diagnostics; conventional PCR (cnPCR) can still show comparable efficiency^24^ The cnPCR was found to detect SARS-CoV-2 with an adequate yield as an alternative to RT-PCR^25^. For the guaranteed presence of the virus, the cnPCR is used in combination with the Sanger sequencing. Spike-gene specific amplification following Sanger sequencing of representative cnPCR positive samples thus confirmed the presence of virus among the contradictory samples (false-positive and false-negative). Spike gene amplification was considered since it is the second-highest expressed gene and is also less mutating than the N gene^26^. A small amplicon (228 bp) was targeted to identify even the fragmented RNA genome due to sampling issues and shorter transcripts. Besides, the cnPCR worked well in some cases where the probe-based method failed due to fluorescence-blocking inhibitors^27^. We determined 17 instances for the Sansure negative samples showing positive or undetermined results in the cnPCR (Table 2).

Standard curve analysis showed a slightly less correlation and efficiency than desired for E gene (slope = − 3.0, R^2^ = 0.98) in 10^0^–10^–5^ dilution series. The reason might be the presence of non-specific RNA after extraction and inhibitors with extracted RNA, presence of E gene at low concentration, and primer-dimer formation. The values for the N gene fell within the standard range (slope = − 3.55, R^2^ = 0.99) as it is present in a higher number than the E gene.

Our target is not to quantify instead to detect the presence or absence of virus in the clinical samples that we have accomplished here. Nevertheless, this study has some limitations. In melt curve analysis, the dominance of N and E gene presence was observed in column-based and crude RNA extraction, respectively. Other researchers, however, reported a similar trend despite working with a very small percentage of the samples^28^. Using a lower amount of RNA template, multiplexing of three genes in SYBR Green, and arises of possible mutations during the study in highly dynamic nucleocapsid protein^26,28^ might reduce the chance to amplify N gene-specific region. Moreover, the sensitivity and the specificity were not wholly determined because of not validating all the studied samples further in cell culture. However, we endeavored to determine an identical sensitivity and specificity while comparing the deviated samples between SYBR Green and Sansure assay (Table 2). There is a huge chance to recover lower concentrated RNA in column-based extraction after elution. Still, in the quick extract method, the recovery rate of lower concentrated RNA is low, giving false-negative results (Table 2)^14^. Another subtle limitation of our technology is that only expert personnel will analyze the results since it is based on melting curve analysis. Finally, PCR/Sanger-based validation was found more accurate when the Ct value was less (usually < 30), and false-positive cases would be higher if the PCR test was used as the sole gold standard.

SYBR Green technique was suitable for detecting SARS-CoV-2 in clinical samples; and our user-friendly and affordable protocol detected clinical SARS-CoV-2 as efficiently as the standard costly Taqman protocol. Since this SYBR green-based method of SARS-CoV-2 detection was performed well after optimization and the results can be easily predicted by melting curve analysis, we recommend this method as an easier, cheaper, and reliable alternative to the costly probe-based method to increase the testing capacity for low- and middle-income countries where reagent supply is limited and high testing capacity is desired.

## Methods

### Primer designing for SARS-CoV-2 strains

We designed our primers by targeting N, E, S, and RdRp protein coding nucleotide sequences based on the aligned sequence data of all circulating SARS-CoV-2 genome sequences available in GISAID. We used CoVariants (https://covariants.org/variants) and National Genomics Data Center (https://bigd.big.ac.cn/ncov/variation/annotation) for checking whether the mutation sites fall within the primer binding region or not. We also checked the primers against the genome of *Homo sapiens*, six common human coronaviruses HCoV-OC43, HCoV-229E, HCoV-NL63, HCoV-HKU1, MERS-CoV, and SARS-CoV, and finally main respiratory and opportunistic viruses and pathogens in PrimerBLAST (Supplementary Table S1). As the internal control, we primarily targeted three house-keeping genes of the human genome, GAPDH and beta-actin. We designed the SARS-CoV-2 specific primers in a way to maintain the annealing temperature at ~ 60 °C for efficient qPCR detection and then used oligo-analyzer tool to check the primers for stem-loops and highly energetic dimer formation (< − 9 kcal/mol). The amplicons’ size was distinct so that we could identify separate products in the melting curve (Table 1).

### Clinical sample selection and ethical consideration

The sample size was calculated based on the prevalence of positive samples using the following equation

$$Sample\,\, Size= \frac{{Z Score}^{2}\times Standard \,\,Deviation\times (1-Standard\,\, Deviation)}{({Margin \,\,of \,\,error)}^{2}}$$

Randomly (random number generator using Microsoft Excel inc.) selected 6 positive and 4 negative samples were selected from left-over samples once in a week and tested by our proposed method besides the routine TaqMan based method. In 9 weeks from 10 October 2020 to 30 November 2020, we collected a total sample size of 3836 where 560 samples were positive. Using the above equation with a 10% margin of error and 95% confidence level for the samples and finally a total of 90 nasopharyngeal swab samples (sample set A) were selected. Similarly, another set of 90 samples (sample set B) was collected in between August and September of 2021 from 12,154 samples (2330 positive cases). All patients’ samples were selected from the continuous surveillance of COVID-19 at the Genome Center, Jashore University of Science and Technology (JUST). The study was approved by the ethical review committee (ERC) of Jashore University of Science and Technology, Bangladesh (Reference: ERC/FBS/JUST/2020-45, Date: 06/10/2020). We performed all experiments according to the relevant guidelines and regulations. The participants were informed about the study, provided their informed consent, and we used their left-over samples from the routine surveillance.

### Crude RNA extraction

According to the manufacturer's instruction, the viral RNA was extracted from the sample set’A (Appendix 1) using QuickExtract™ RNA Extraction Kit (Lucigen, USA) (cat number: QER090150). Briefly, a particular volume of the samples (5–100 µl) VTM was separated from patients swab sample inside Biosafety Cabinet (Class II) and mixed with an equal volume of ice-cold QuickExtract RNA Extraction Solution (5–100 µl) in Eppendorf tube and vortex-mixed for 1 min, followed by immediate transfer into ice.

### Column based RNA extraction

To better ensure our method compared to the commercial kit, we extracted RNA from sample set B (Appendix 2) using a more efficient column-based RNA extraction system using AFCPrep™ Viral RNA extraction Kit (cat. AFC-VRNA-048), according to the manufacturer’s instructions. Briefly, after vortexing the sample tube, we transferred 140 µl of sample into a nuclease-free Eppendorf tube and then we added 560 µl of AFCL buffer, vortexed, and incubated at room temperature for 10 min. After that, we added 560 µl ethanol (96 ~ 100%) in the tube and mixed well by brief vortexing. Afterward, we combined a RNA extraction (RE) Column with a Collection Tube and transferred up to 700 µl of sample mixture to the RE Column, centrifuged for 1 min at 12,000 RPM, discarded the flow-through, and transferred the remaining sample mixture to the RE Column and centrifuging under the same conditions as before. We then discarded the flow-through, and reused the Collection Tube. Then, we performed similar steps with Wash Buffer-1(500 µl), Wash Buffer-2 (700 µl, washed twice), and blank RE Column to dry. Finally, we combined the RE Column with a new Elution Tube, added 55 µl of RNase-free water, and centrifuged at 15,000 RPM for 3 min to elute the viral RNA.

### Commercial fluorescence-based RT-PCR

In TaqMan probe-based RT-qPCR method, commercially available SARS-CoV-2 nucleic acid detection kits (Sansure Biotech, China) were compared with the in-house SYBR Green kit. The kit contains SARS-CoV-2 ORF1ab and N genes, and as internal control, human IRC genes (i.e., Rnase P). The reaction conditions and procedures were applied according to the protocol described elsewhere and all reactions were performed in duplicate to confirm reproducibility. The reaction systems and methods were carried out according to the instructions of the kits (http://eng.sansure.com.cn/index.php?g=&m=article&a=index&id=81). In brief, 13 μl 2019-nCoV-PCR Mix was mixed with 2 μl 2019-nCoV-PCR-Enzyme Mix and added to 10 μl template RNA. The RT-qPCR conditions were set according to the manufacturer’s instructions as follows: reverse transcription at 50 °C for 30 m’nutes, cDNA pre denaturation at 95 °C for 1 min, then denaturation at 95 °C for 15 s and annealing, extension and fluorescence collection at 60 °C for 30 s for 45 cycles before cooling the device at 25 °C for 10 s in Real Time PCR machine (QuantStudio 3.0, Applied Biosystem). For detection, FAM, ROX, and VIC were used to detect ORF1ab, N gene, and internal control, respectively. Supplied Positive control and supplied negative control with kit as well as nuclease-free water was included in every qPCR run as a positive control, kit negative control and reaction negative control, respectively. Sigmoid curve for either or both of the ORF1b-nsp14 or N gene with a CT value of ≤ 36 was interpreted as positive. CT values between 37 and 39 were repeated and above those (≥ 40) were considered negative in the prevalence study.

### Optimization of singleplex RT-PCR

To detect SARS-CoV-2 target genes, melting curve-based RT-PCR was performed using SYBR Green fluorescent dye, which binds double-stranded DNA by intercalating between the DNA bases. All RT‐qPCRs were performed on Applied Biosystems QuantStudio3 Real‐Time PCR Systems and Design and Analysis Software v1.5.1, using 0.2 ml MicroAmp™ Optical 96-Well Reaction Plate (Cat. No. N8010560) and MicroAmp™ Optical Adhesive Film (Cat. No. 4311971). Initially, commercial kit based repeatedly confirmed positive and negative samples were considered to check the accuracy and efficiency of each primer set. We verified this through one step RT-qPCR amplification (QuantStudio 3.0: Applied Bioscience) of the amplicons to optimize the PCR conditions and primer set concentration. The target SARS-CoV-2 genes included N1, S1, E1, and RdRp1. In addition, either GAPDH or β-actin of humans, a housekeeping gene, was used as an internal positive control. For each reaction, 5 μl of extracted RNA template was used for the SARS-CoV-2 specific target primer sets and human internal positive control primer set. Gradient RT-qPCR was performed with an increasing annealing temperature (Tm) from 60 to 70 °C based on the melting temperature of each primer set. Different primer concentrations (forward, reverse; 50 nM, 75 nM; 100 nM, 150 nM; 200 nM, 250 nM)were used to optimize the engagement of the primer sets.

The basic RT-qPCR conditions were set according to the Luna® Universal SYBR green One-Step RT-qPCR Kit (New England Biolabs Inc, MA). Detailed protocol is provided in the manufacturer’s recommended procedures (https://international.neb.com/products/e’005-luna-universal-one-step-rt-qpcr-kit#Product%20Information). We mixed 10 μl master mix (2X) with 1 μl enzyme mix (20X), 200 nM forward primer, and 250 nM reverse primer for each gene in single primer set. The PCR conditions were set at 58 °C incubation for 12 min for reverse transcription followed by the initial denaturation at 95 °C for 3 min, then cycle denaturation at 95 °C for 15 s and an extension at 62 °C for 25 s for 45 cycles. For the melt curve analysis, we set 95 °C for 15 s, then 60 °C for 1 min followed by 95 °C for 15 s. The ramp rate of last transformation of 60 °C to 95 °C was set at 0.05 °C/seconds. For passive reference, ROX was used as the passive reference and SYBR dye was used to check the fluorescence in the Real Time PCR machine (QuantStudio 3.0, Applied Biosystem). Low Ct valued samples (< 20 as detected in TaqMan method) and nuclease-free water were included in every qPCR run as a positive control and negative control, respectively.

### Optimization of multiplex RT-PCR

For the multiplexing, two sets of SARS-CoV-2 specific primers, JUST_N1 and JUST_E1, as well as, β-actin (human control) primer set were used since the amplified products had distinct melting curve peaks—that eased the identification of different products. The primer concentration was optimized for multiplex RT-qPCR as follows: JUST_N1 forward 300 nM, JUST_N1 reverse 400 nM, JUST_E1 forward 150 nM, JUST_E1 reverse 175 nM, β-actin forward 150 nM, and β-actin reverse 200 nM. 10 μl master mix (2×) with 1 μl enzyme mix (20X) of Luna® Universal One-Step RT-qPCR Kit (New England Bioscience) with 5 μl of template RNA was used under the PCR conditions as performed for the singleplex one. We set the criteria to determine the positive and negative results: presence of any peak for either N or E genes in the melting curve. The results were not interpreted based on the derivative reporter value of the melting curve for each gene since a peak with low value but good shape was considered in this case. The overall protocol of the assay has been presented within a single kit-based manual style (Fig. 3).

### Generation of standard curve and melting curve analysis

For preparing the standard curve, template RNA was diluted tenfold for 5 times (10^0^ to 10^–5^). The diluted template RNA was used to conduct both single-plex RT-qPCR assay for JUST_N1 and JUST_E1. The derivative reporter values of the melting curve as measured for the N and E genes were analyzed to compare between the SYBR positive-Sansure positive and SYBR positive- Sansure negative groups. We targeted to set a threshold based on the derivative reporter value from the melting curve.

### Assay precision and validation of RT-PCR method

We performed technical replicates (triplicate) for the samples that were extracted by column purification method. To ensure amplification of the correct RT-PCR products, we performed gel electrophoresis in 3% agarose gel having ethidium bromide at 60 V and 100 mA for 100 min and analyzed in an automated Gel Doc Imager (Molecular Imager® Gel Doc™ XR + System with Image Lab™ Software by Bio-Rad [Catalog # 170-8195] and the software Image Lab™ Software version 5.2.1). To re-validate the contradictory results between the SYBR-Green and Sansure assay, the in-house established primer set targeting the spike gene for amplification established by Islam et al. (2021) was used and run in 1.5% agarose gel at 80 V and 200 mA for 40 min to ensure amplification^29^.

### Automated RNA extraction protocol for the contradictory samples

For the contradictory samples (false-positive and false-negative), we here used automated RNA extraction for better efficiency. MagMAX™ Viral/Pathogen Nucleic Acid Isolation Kit (Cat. No. A42352) for better extraction of contradictory samples according to the manufacturer's instructions. Briefly, we added 10 µl Proteinase K, 1000 µl Wash buffer, 1000 µl 80% ethanol, and 500 µl 80% ethanol to each well of different rows of the sample plate. Afterwards, we added 200 µl sample and 550 µl Binding Bead Mix (530 µl Binding Solution + 20 µl Total Nucleic Acid Magnetic Beads) into wells of a row A and tip comb in the last row of the sample plate. Besides, we added 40 µl Elution solution to each well of the elution strip. Selecting the program MVP_DUO on the instrument KingFisher™ Duo Prime and starting the run, then loaded the Elution Strip and Sample Plate into position when prompted by the instrument. After the protocol was complete (~ 25 min after start), we immediately removed the Elution Strip from the instrument and recovered RNA elute.

### Sanger sequencing of the amplified products (spike gene)

For purifying the desired PCR product from the low melting 1% agarose gel, we used Wizard® SV gel and PCR Clean-Up system. The representative amplicons were then subjected to Sanger sequencing with BigDye™ Terminator v3.1 Cycle Sequencing Kit (Thermo Fisher Scientific) in Applied Biosystems SeqStudio genetic analyzer as per the optimized protocol of Islam et al. (2021). The trace or chromatogram (.ab1) files from the Sanger sequencing were analyzed using the Sequencing Analysis Software V6.0 (Thermofisher, USA). NCBI BLAST was performed initially and the alignment to SARS-CoV-2 spike gene was also checked in MEGA7 (https://www.megasoftware.net/).

### Statistical analysis

Sensitivity, Specificity, kappa index, Positive predictive value (PPV) and Negative predictive value (NPV) were calculated by analyzing data in IBM SPSS Statistics. Positive Likelihood ratio (LR +) and Negative likelihood ratio (LR-) were calculate using MedCalc Software Ltd. Diagnostic test evaluation calculator. https://www.medcalc.org/calc/diagnostic_test.php (Version 20.027; accessed February 16, 2022). Simple linear regression analysis and standard curve were generated RStudio using packages: readxl, ggplot2, ggpubr and ggpmisc.

## Supplementary Information

Supplementary Information 1.

Supplementary Information 2.

Supplementary Information 3.

Supplementary Information 4.

## References

[1] Bogoch II (2020). Pneumonia of unknown aetiology in Wuhan, China: potential for international spread via commercial air travel. J. Travel Med..

[2] Vogels CBF (2020). Analytical sensitivity and efficiency comparisons of SARS-CoV-2 RT–qPCR primer–probe sets. Nat. Microbiol..

[3] Corman VM (2020). Detection of 2019 novel coronavirus (2019-nCoV) by real-time RT-PCR. Eurosurveillance.

[4] Smithgall MC, Dowlatshahi M, Spitalnik SL, Hod EA, Rai AJ (2021). Types of assays for SARS-COV-2 testing: A review. Lab Med..

[5] Tajadini M, Panjehpour M, Javanmard SH (2014). Comparison of SYBR Green and TaqMan methods in quantitative real-time polymerase chain reaction analysis of four adenosine receptor subtypes. Adv. Biomed. Res..

[6] Marinowic DR (2021). A new SYBR Green real-time PCR to detect SARS-CoV-2. Sci. Rep..

[7] conventional RT-PCR and SYBR Green-based RT-qPCR (2020). Gustavo Dorlass, E. *et al.* Lower cost alternatives for molecular diagnosis of COVID-19. Braz. J. Microbiol..

[8] Dharavath, B. *et al.* A one-step, one-tube real-time RT-PCR based assay with an automated analysis for detection of SARS-CoV-2. *Heliyon* **6**, e04405 (2020). 10.1016/j.heliyon.2020.e04405 PMC7341355 32665985

[9] Ganguly, D. R. *et al.* SYBR green one-step qRT-PCR for the detection of SARS-CoV-2 RNA in saliva. *bioRxiv* 1 (2020). 10.1101/2020.05.29.109702.

[10] Park M, Won J, Choi BY, Lee CJ (2020). Optimization of primer sets and detection protocols for SARS-CoV-2 of coronavirus disease 2019 (COVID-19) using PCR and real-time PCR. Exp. Mol. Med..

[11] Won J (2020). Development of a laboratory-safe and low-cost detection protocol for SARS-CoV-2 of the coronavirus disease 2019 (COVID-19). Exp. Neurobiol..

[12] Pereira-Gómez, M. *et al.* Evaluation of SYBR Green real time PCR for detecting SARS-CoV-2 from clinical samples. *J. Virol. Methods* **289**, 114035 (2021). 10.1016/j.jviromet.2020.114035 PMC7831559 33285190

[13] Hernández C (2021). Evaluation of the diagnostic performance of nine commercial RT-PCR kits for the detection of SARS-CoV-2 in Colombia. J. Med. Virol..

[14] Ali, N., Rampazzo, R. de C. P., Costa, A. D. T. & Krieger, M. A. Current nucleic acid extraction methods and their implications to point-of-care diagnostics. *Biomed Res. Int.* **2017**, 9306564 (2017). 10.1155/2017/9306564 PMC5529626 28785592

[15] Kim D (2020). The architecture of SARS-CoV-2 transcriptome. Cell.

[16] Eskier, D., Karakülah, G., Suner, A. & Oktay, Y. RdRp mutations are associated with SARS-CoV-2 genome evolution. *PeerJ* **8**, (2020). 10.7717/peerj.9587 PMC7380272 32742818

[17] Chan, P. K. S. & Chan, M. C. W. Tracing the SARS-coronavirus. *J. Thorac. Dis. Vol 5, Suppl. 2 (August 2013) J. Thorac. Dis. (Emerging Infect. Respir. Dis. & 10th Anniv. SARS Epidemics)* (2013). 10.3978/j.issn.2072-1439.2013.06.19 PMC3747522 23977431

[18] Centre for Disease Control and Prevention. SARS-CoV-2 Variant Classifications and Definitions. *Cdc* https://www.cdc.gov/coronavirus/2019-ncov/cases-updates/variant-surveillance/variant-info.html#Interest (2021).

[19] Bernard PS, Pritham GH, Wittwer CT (1999). Color multiplexing hybridization probes using the apolipoprotein E locus as a model system for genotyping. Anal. Biochem..

[20] Tanaka J (2019). KRAS genotyping by digital PCR combined with melting curve analysis. Sci. Rep..

[21] Budiarto BR, Harahap WA, Desriani D (2016). Development of Sybr green I-based melting curve method for HER2I655V polymorphism detection in breast cancer. Makara J. Heal. Res..

[22] Sidstedt M, Rådström P, Hedman J (2020). PCR inhibition in qPCR, dPCR and MPS—mechanisms and solutions. Anal. Bioanal. Chem..

[23] Lu, Y. *et al.* Comparison of the diagnostic efficacy between two PCR test kits for SARS-CoV-2 nucleic acid detection. *J. Clin. Lab. Anal.* **34**, e23554 (2020). 10.1002/jcla.23554 PMC7536918 32977349

[24] Patrick, B., W., P. G. & Udo, R. Quantitative real-time PCR is not more sensitive than “conventional” PCR. *J. Clin. Microbiol.* **46**, 1897–1900 (2008). 10.1128/JCM.02258-07 PMC2446855 18400914

[25] Carvalho, R. F. *et al.* Validation of conventional PCR-like alternative to SARS-CoV-2 detection with target nucleocapsid protein gene in naso-oropharyngeal samples. *PLoS One* **16**, e0257350 (2021). 10.1371/journal.pone.0257350 PMC8459981 34555073

[26] Rahman MS (2021). Evolutionary dynamics of SARS-CoV-2 nucleocapsid protein and its consequences. J. Med. Virol..

[27] Schrader C, Schielke A, Ellerbroek L, Johne R (2012). PCR inhibitors—occurrence, properties and removal. J. Appl. Microbiol..

[28] Hasan MR (2021). A novel point mutation in the N gene of SARS-CoV-2 may affect the detection of the virus by reverse transcription-quantitative PCR. J. Clin. Microbiol..

[29] Islam MT (2021). A rapid and cost-effective multiplex ARMS-PCR method for the simultaneous genotyping of the circulating SARS-CoV-2 phylogenetic clades. J. Med. Virol..

