# Supplementary material for: Development and validation of cost-effective one-step multiplex RT-PCR assay for detecting the SARS-CoV-2 infection using SYBR Green melting curve analysis
Source: Sci Rep. 2022 Apr 20;12:6501. doi: 10.1038/s41598-022-10413-7 (PMC9019801; doi:10.1038/s41598-022-10413-7)
Supplement: Supplementary file 4 — Supplementary Information 4. [file 41598_2022_10413_MOESM4_ESM.pdf]

Supplementary Table S1: Designed primers for this study and the mutations profile link.

Primer name	Mutation in 3' end	Specificity of Primer (*Homo Sapiens*, other organisms*)	https://covariants.org/variants	https://bigd.big.ac.cn/ncov/variation/annotation
JUST_E1_F	No[$]	No target templates were found in selected database: Refseq mRNA (Organism limited to *Homo sapiens*)	No Mutation (at the 4th base of 3´-end[#]**)**	26270, 26256, 26262, 26257
JUST_E1_R	No[$]	Target template found with SARS-CoV-2 isolate Wuhan-Hu-1 and SARS coronavirus Tor2, complete genome	No Mutation[$]	None
JUST_N1_F	No[$]	No target templates were found in selected database: Refseq mRNA (Organism limited to Homo sapiens)	No Mutation[$]	29422, 29427,
JUST_N1_R	No[$]	Target template found with SARS-CoV-2 isolate Wuhan-Hu-1 and SARS coronavirus Tor2, complete genome	No Mutation[$]	28541
JUST_RdRp1_F	No	No target templates were found in selected database: Refseq mRNA (Organism limited to Homo sapiens)	No mutation	None
JUST_RdRp1_R	No	Target template found with SARS-CoV-2 isolate Wuhan-Hu-1 and SARS coronavirus Tor2, complete genome	No mutation	None
JUST_S1_F	No	No target templates were found in selected database: Refseq mRNA (Organism limited to Homo sapiens)	No mutation	21637, 21638, 21621, 21624
JUST_S1_R	No	Target template found with SARS-CoV-2 isolate Wuhan-Hu-1	No mutation	23664
		*Organisms investigated (Taxonomy ID): HCoV-HKU1 (290028); HCoV-OC43 (31631); HCoV-NL63 (277944); HCoV-229E (11137); MERS-CoV (1335626); H1N1 (114727); H3N2 (119210); Influenza (untyped) (11309); H5N1 (102793); H7N9 (333278); Influenza B (11520); Respiratory Syncytial Virus (11250); Parainfluenza 1 virus (12730); Parainfluenza 2 virus (1979160); Parainfluenza 3 virus (11216); Parainfluenza 4 virus A (11224); Parainfluenza 4 virus B (11226); Human Metapneumovirus (162145); Human Bocavirus (329641); Mycoplasma spp. (2093). Rhinovirus/Enterovirus (12059); SARS-CoV		

$ Considering mutations of Delta and Omicron variants

For the omicron variant E:T9I (C26T) mutation.

Supplementary Table S2: Data of primer optimization in different combinations

Primer Combination	Sample ID	Ct Value	Melting Temperature	Derivative Reporter	
**Single-plex Assay**					
JUST_N1	103.49	28.865	82.117	49,427.047	JUST_N1 Specific (Positive)
	103.59	27.081	82.422	44,041.594	
	105.92	24.958	82.422	33,674.445	
	105.82	31.496	74.949	33,807.930	Non-specific (Negative)
			80.897	16,299.283	
JUST_E1	103.49	28.266	79.046	40,590.711	JUST_E1 Specific (Positive)
	103.59	26.847	79.504	50,604.645	
	105.92	25.883	79.656	48,298.816	
	105.82	33.038	66.095	10,334.008	Non-specific (Negative)
			82.246	20,230.045	
			85.141	17,941.426	
JUST_S1	103.49	25.997	76.324	51,881.965	JUST_S1 Specific (Positive)
	103.59	25.233	76.629	34,492.000	
	105.92	23.581	76.629	36,840.020	
	105.82	31.273	67.628	9,944.588	Non-specific (Negative)
			74.341	30,616.861	
JUST_RdRp1	103.49	27.599	77.366	50,752.266	JUST_RdRp1

	103.59	26.594	77.671	50,150.043	Specific (Positive)
	105.92	24.918	77.671	41,050.215	
	105.82	34.294	80.413	28,342.781	Non-specific (Negative)
Beta-actin	117.199	23.377	87.728	51,152.402	Beta-actin Specific
	100.115	25.559	87.470	49,336.742	
GAPDH	117.199	31.161	82.928	63,028.238	GAPDH Specific
			69.481	17,801.490	Non-specific
	100.115	36.145	82.979	45,427.840	GAPDH Specific
**Duplex Assay**					
JUST_N1 +JUST_E1	103.59	25.944	80.899	18,224.209	JUST_N1 Specific
			77.696	9,732.452	JUST_E1 Specific
	117.199	24.647	82.278	31,206.523	JUST_N1 Specific
			78.905	97,134.820	JUST_E1 Specific
JUST_N1 +JUST_S1	103.59	26.613	81.662	16,774.512	JUST_N1 Specific
			75.408	11,924.494	JUST_S1 Specific
	117.199	24.178	82.278	16,230.109	JUST_N1 Specific
			75.788	54,621.520	JUST_S1 Specific
JUST_N1 +JUST_RdRp1	103.59	26.774	81.967	18,510.281	JUST_N1 Specific
			77.086	19,971.842	JUST_RdRp1 Specific
	117.199	22.934	82.227	25,829.594	JUST_N1 Specific
			76.861	67,849.789	JUST_RdRp1

					Specific
JUST_E1 +JUST_S1	117.199	24.265	78.956	80,005.727	JUST_E1 Specific
JUST_E1 +JUST_RdRp1	117.199	23.429	78.905	61,383.828	JUST_E1 Specific
			76.605	25,566.580	JUST_RdRp1 Specific
JUST_S1 +JUST_RdRp1	117.199	22.878	77.014	68,878.430	JUST_RdRp1 Specific
JUST_N1 +GAPDH	117.199	24.509	82.267	51,459.141	JUST_N1 Specific
			83.905	29,276.434	GAPDH Specific
			73.361	14,140.837	Non-specific
JUST_E1 +GAPDH	117.199	27.825	78.889	62,769.035	JUST_E1 Specific
			82.421	23,866.477	GAPDH Specific
JUST_S1 +GAPDH	117.199	24.897	75.818	48,897.164	JUST_S1 Specific
			82.421	14,717.176	GAPDH Specific
**Triplex**					
JUST_N1 +JUST_E1 +JUST_S1	103.59	26.525	82.425	12,021.470	JUST_N1 Specific
			76.018	6,761.184	JUST_S1 Specific
JUST_N1	103.59	28.707	82.272	8,179.607	JUST_N1 Specific

+JUST_RdRp1<br>+JUST_S1			77.238	12,854.509	JUST_RdRp1 Specific
JUST_N1<br>+JUST_RdRp1<br>+JUST_E1	103.59	26.544	82.272	10,984.571	JUST_N1 Specific
			79.069	8,844.529	JUST_E1 Specific
JUST_N1<br>+JUST_E1<br>+Beta-actin	117.199	22.999	81.825	29,259.492	JUST_N1 Specific
			78.487	28,967.324	JUST_E1 Specific
			85.780	29,502.145	Beta-actin specific
			87.731	42,329.117	
JUST_N1<br>+JUST_E1<br>+GADPH	117.199	23.226	82.203	56,078.969	JUST_N1 Specific
			78.434	15,534.940	JUST_E1 Specific
**Quadruplex**					
JUST_N1<br>+JUST_E1<br>+JUST_S1<br>+JUST_RdRp1	103.59	26.805	82.120	4,701.885	JUST_N1 Specific
			77.543	9,604.580	JUST_RdRp1 Specific

Table S3. The efficiency values with 95% confidence interval for the assay considering Sansure, as well as, Sansure and S-gene amplification as the gold standard

	Sensitivity	Specificity	PPV	NPV	LR+	LR-	Kappa index
Considering Sansure as standard (n=90)	89.09%<br><br>77.75% to 95.89%	22.86%<br><br>10.42% to 40.14%	64.47%<br><br>59.71% to 68.97%	57.14%<br><br>33.57% to 77.86%	1.15<br><br>0.94 to 1.41	0.48<br><br>0.18 to 1.26	0.134
Considering Sansure & S gene as standard (n=82, except undetermined S gene amplification)	96.67%<br><br>88.47% to 99.59%	54.55%<br><br>32.21% to 75.61%	85.29%<br><br>78.54% to 90.19%	85.71%<br><br>59.31% to 96.11%	2.13<br><br>1.34 to 3.37	0.06<br><br>0.01 to 0.25	0.578
Column based extraction_ Considering Sansure (n=90)	96.83%<br><br>89.00% to 99.61%	92.59%<br><br>75.71% to 99.09%	96.83%<br><br>88.93% to 99.14%	92.59%<br><br>76.09% to 98.00%	13.07<br><br>3.44 to 49.64	0.03<br><br>0.01 to 0.13	0.894
Column based extraction_ Considering Sansure & S gene as standard (n=89)	100.00%<br><br>94.13% to 100.00%	92.86%<br><br>76.50% to 99.12%	96.83%<br><br>88.92% to 99.15%	100.00%	14.00<br><br>3.68 to 53.23	0.00	0.947

Supplementary table S4. Melting curve analysis results

RNA extraction type	Sample types (number)	Both N and E gene-specific melt curve peak	Only N gene-specific melt curve peak	Only E gene-specific melt curve peak
Crude RNA extraction system	Concordant positive samples (49)	22	06	21
	False-positive (SYBR Green positive – Sansure Negative) samples (27)	10	01	16
Column-based RNA extraction system	Concordant positive samples	48	13	0
	False-positive (SYBR Green positive – Sansure Negative) samples	0	02	0

Supplementary Table S5: Cost of the total process per sample

**Reagents and Consumables**	**Cost (per sample)**	**Cost (per sample)**
Lucigen RNA extraction kit	10 BDT ($0.12)	-
Column Based RNA Extraction Kit	-	400 BDT ($4.72)
Luna RT-PCR kit	120 BDT ($1.42)	120 BDT ($1.42)
Primers	3 BDT ($0.03)	3 BDT($0.03)
Consumables	7 BDT ($0.08)	7 BDT($0.08)
Total	140 BDT ($1.65)	530 BDT (~$6)

Supplementary Fig. S1: Gel Electropherogram of RT-PCR amplified products

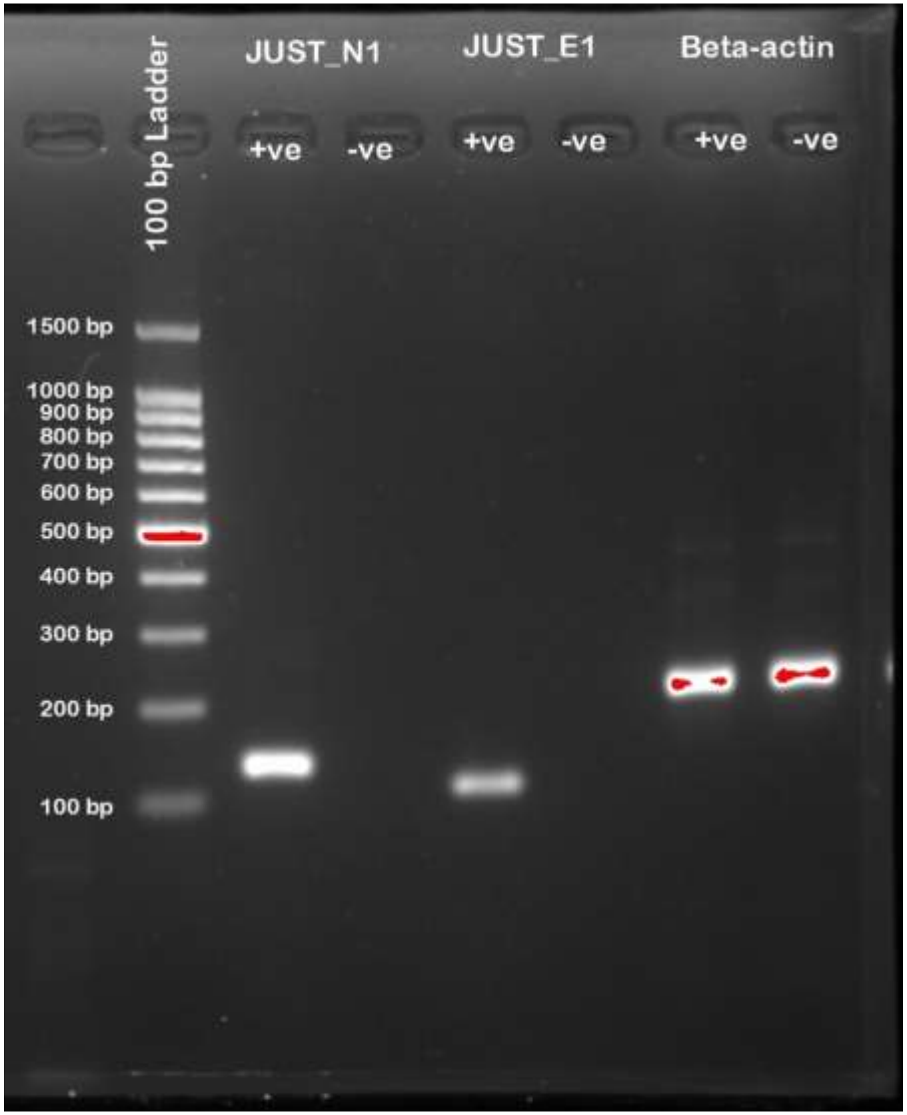

a

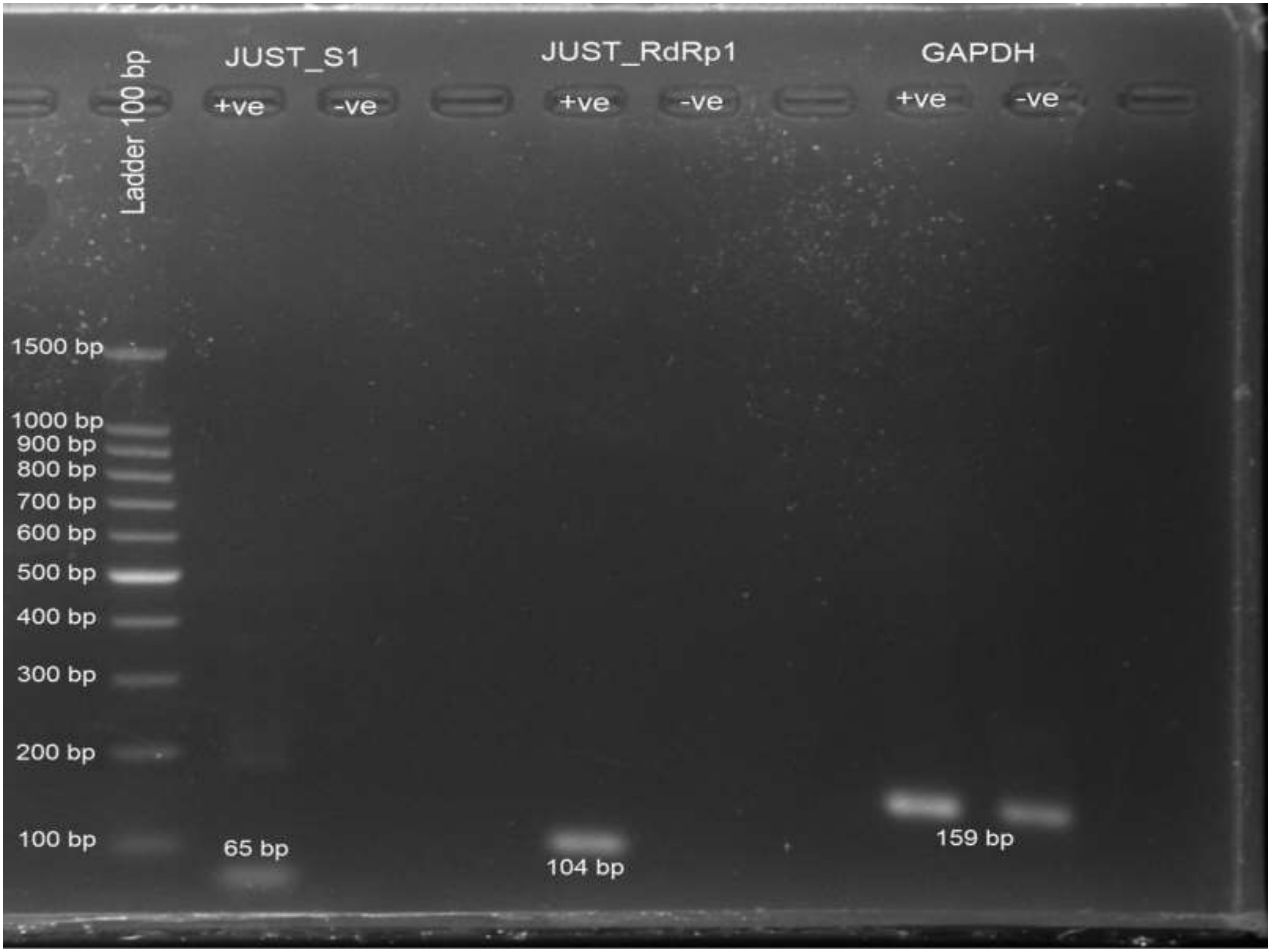
Ladder 100 bp
JUST_S1
+ve
-ve
JUST_RdRp1
+ve
-ve
GAPDH
+ve
-ve
1500 bp
1000 bp
900 bp
800 bp
700 bp
600 bp
500 bp
400 bp
300 bp
200 bp
100 bp
65 bp
104 bp
159 bp

b

Supplementary Fig. S2: melting curve of different genes and final multiplex

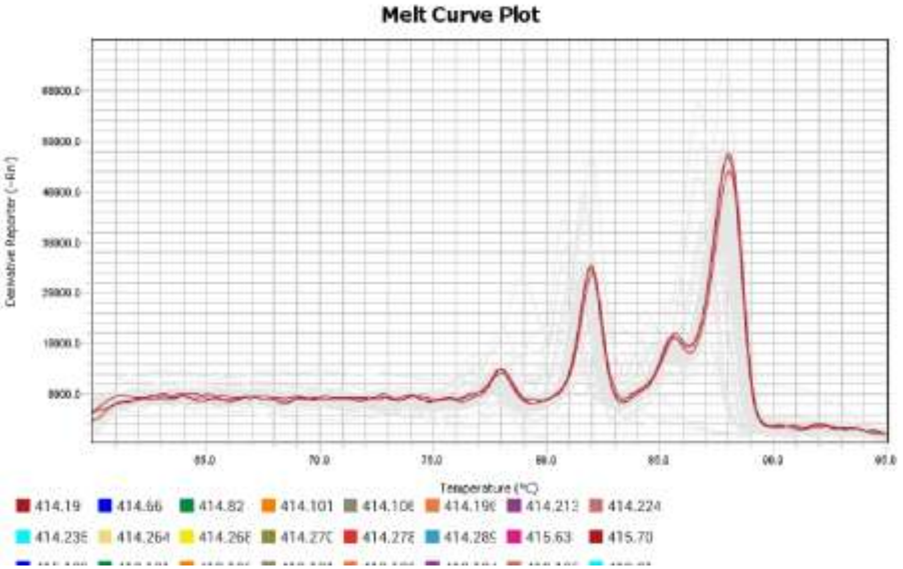 a	Melting curve of positive sample(Triplicate)
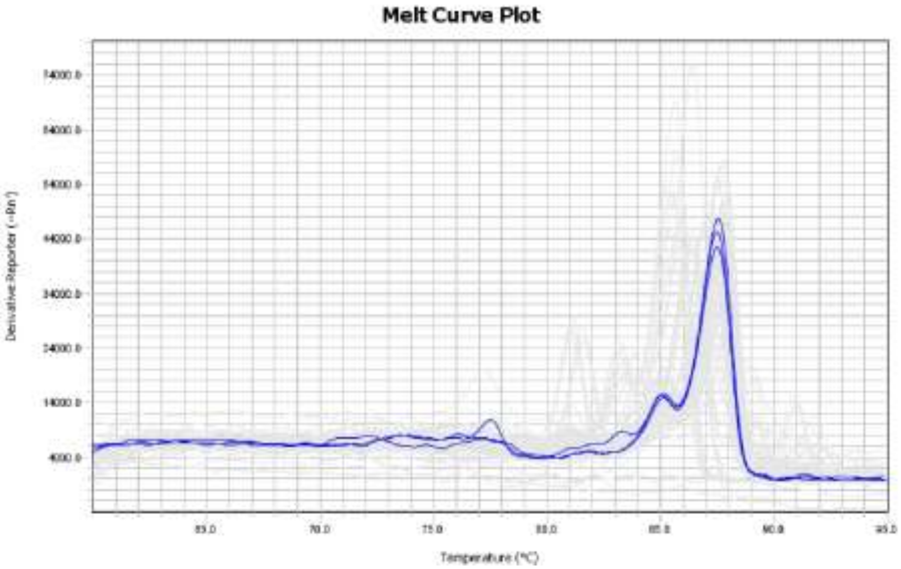 b	Melting curve of negative sample(Triplicate)

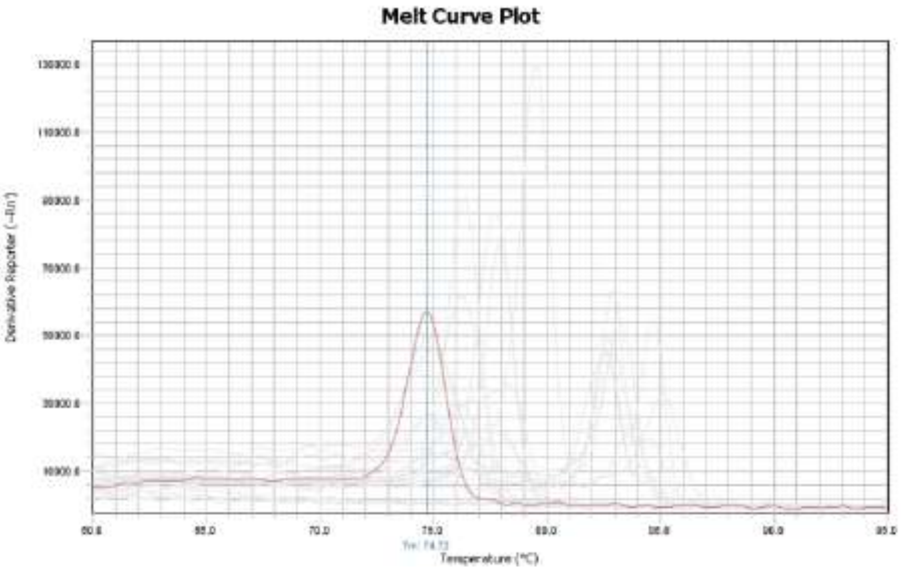 c	Melting curve of positive sample with S gene
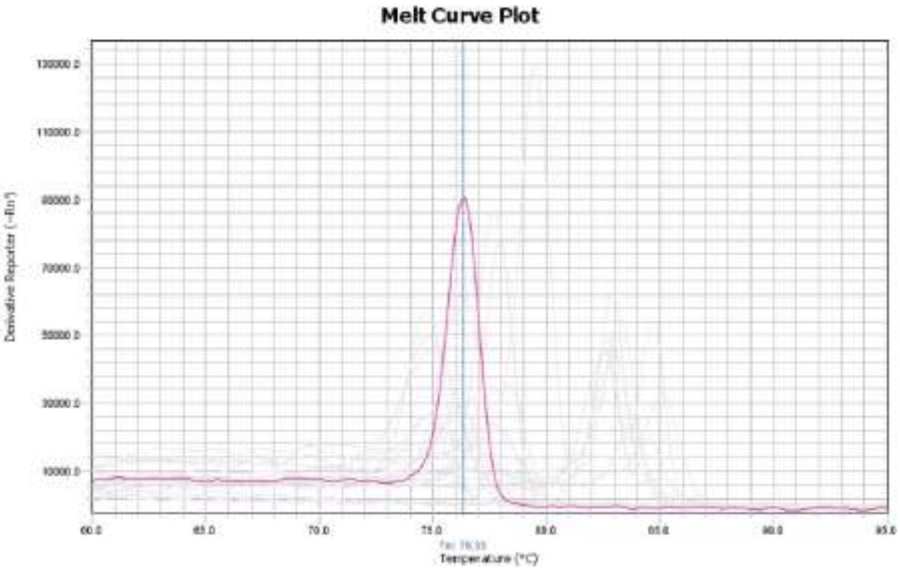 d	Melting curve of positive sample with RdRp gene

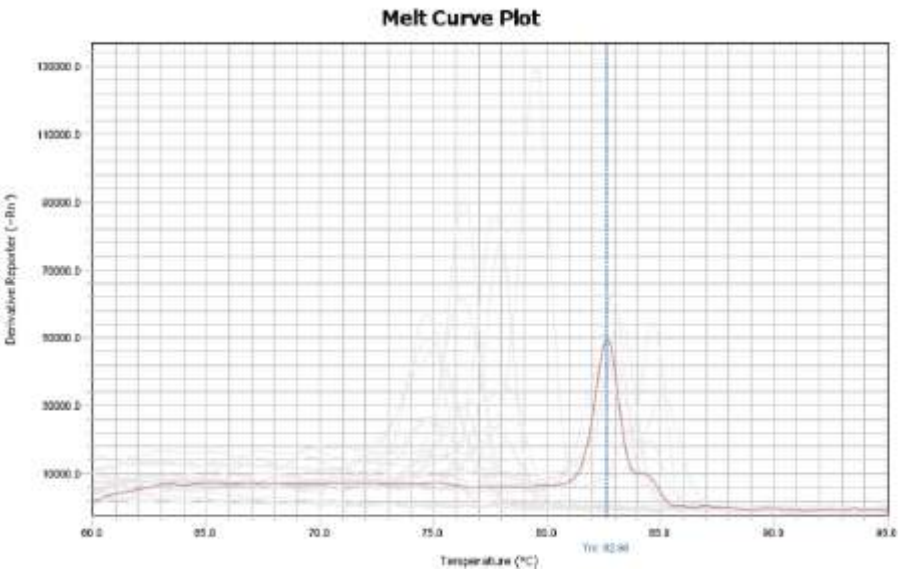<br>**e**	Melting curve of positive sample with GAPDH gene.

Supplementary Fig. S3: Melting curve plot of different genes in different primer combinations.

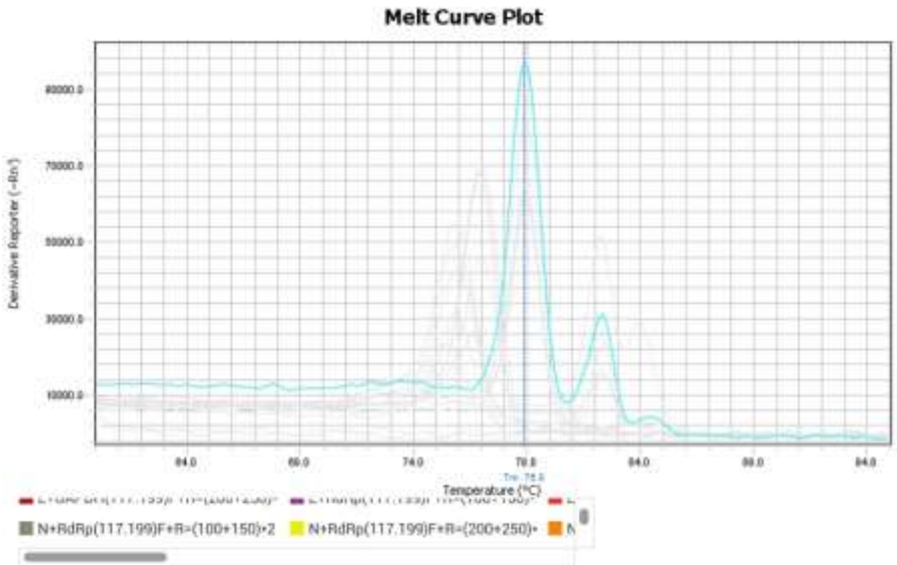	Melt curve plot of the duplex assay using JUST_N1+JUST_E1 primer combination.
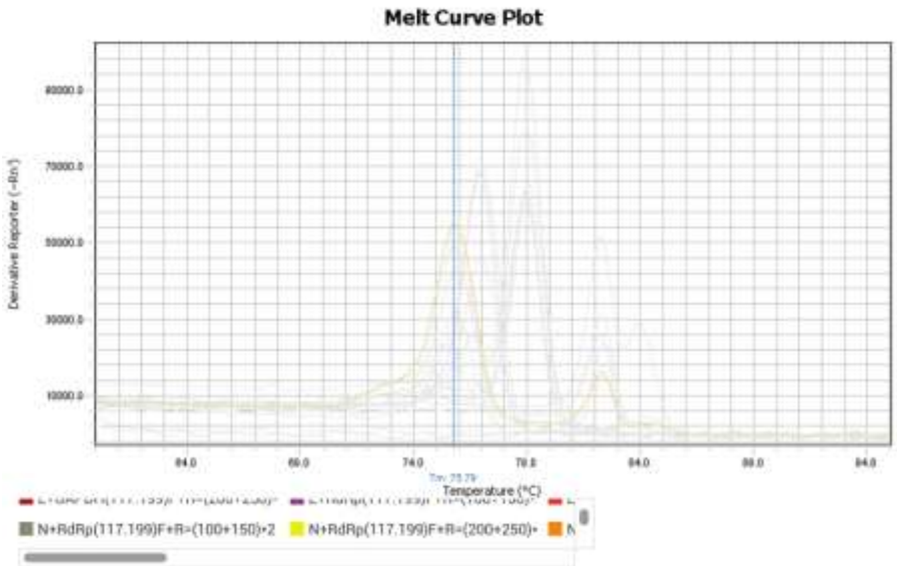	Melt curve plot of the duplex assay using JUST_N1+JUST_S1 primer combination.

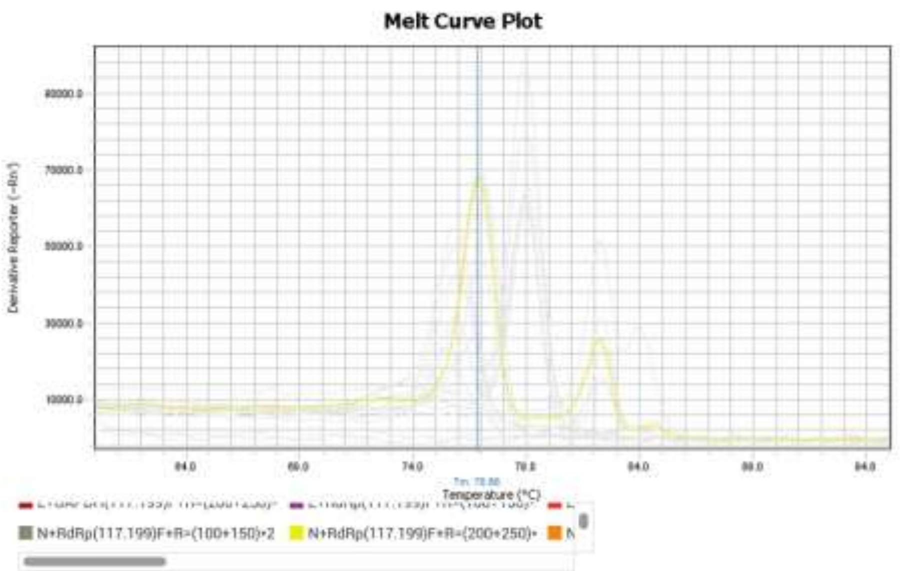	Melt curve plot of the duplex assay using JUST_N1+JUST_RdRp1 primer combination.
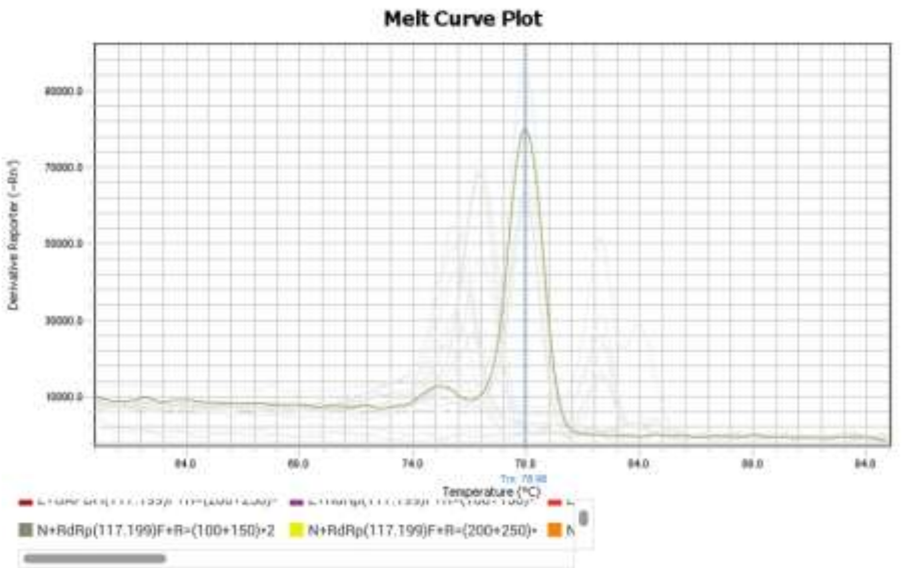	Melt curve plot of the duplex assay using JUST_E1+JUST_S1 primer combination.

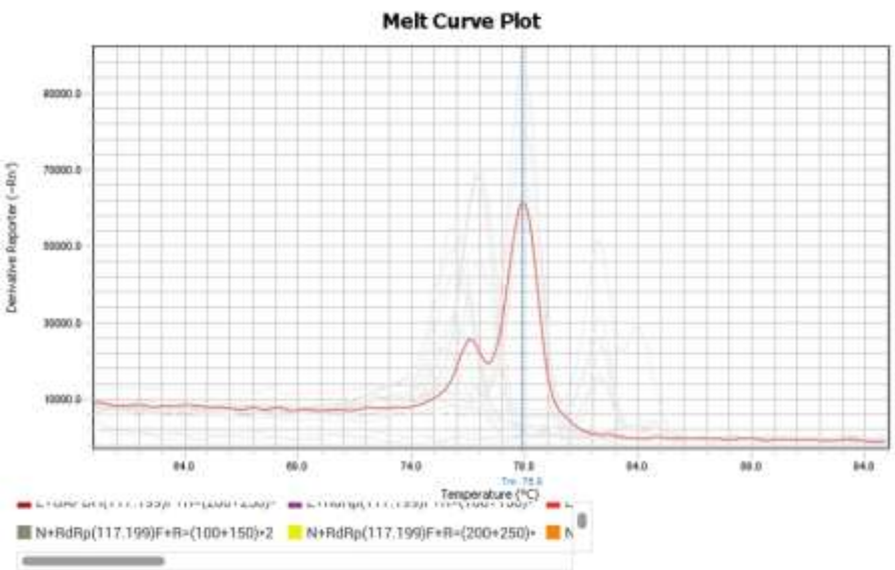	Melt curve plot of the duplex assay using JUST_E1+JUST_RdRp1 primer combination.
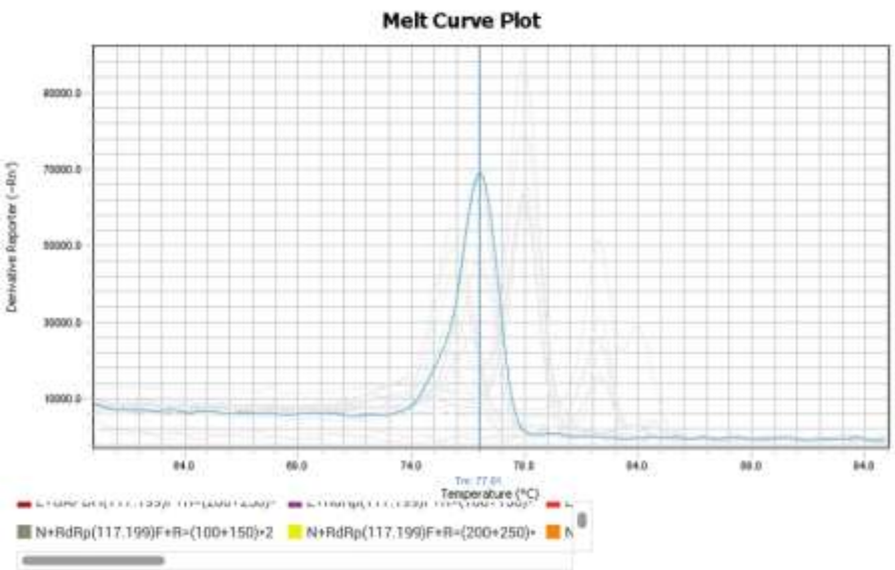	Melt curve plot of the duplex assay using JUST_S1+JUST_RdRp1 primer combination.

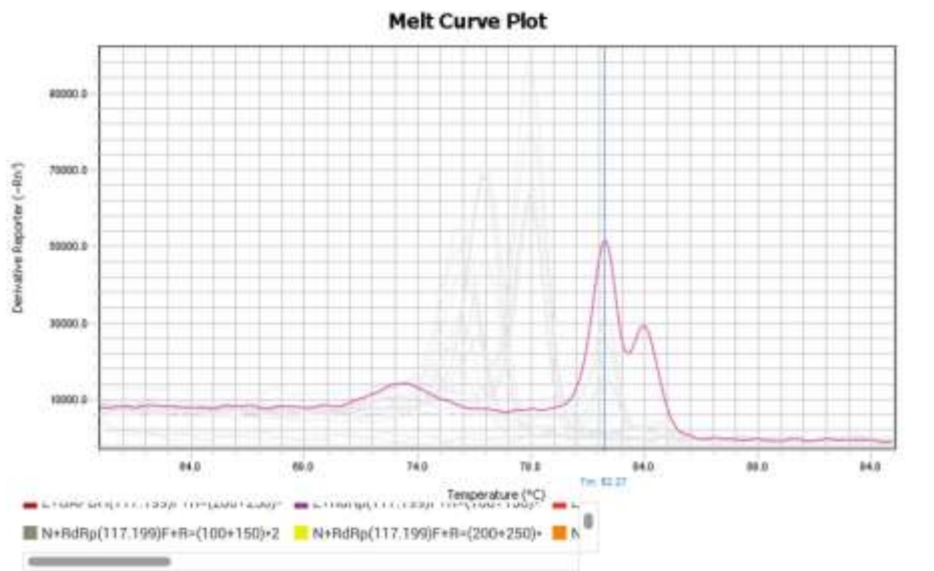	Melt curve plot of the duplex assay using JUST_N1+GAPDH primer combination.
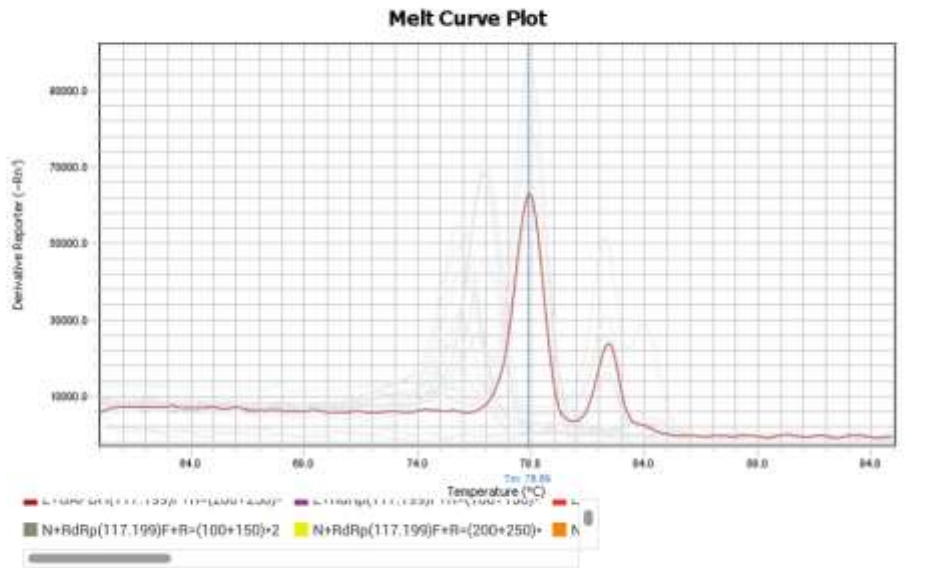	Melt curve plot of the duplex assay using JUST_E1+GAPDH primer combination.

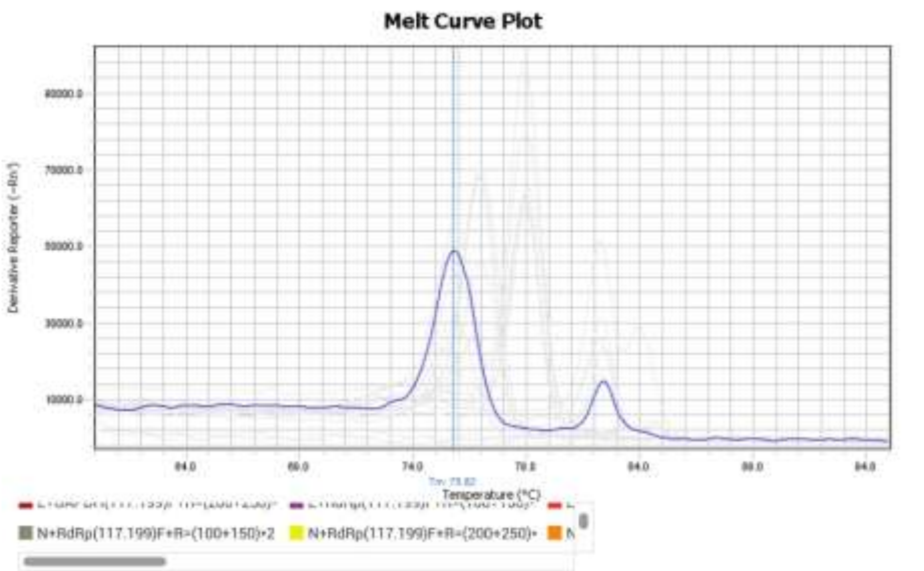

Melt curve plot of the duplex assay using JUST_S1+GAPDH primer combination.

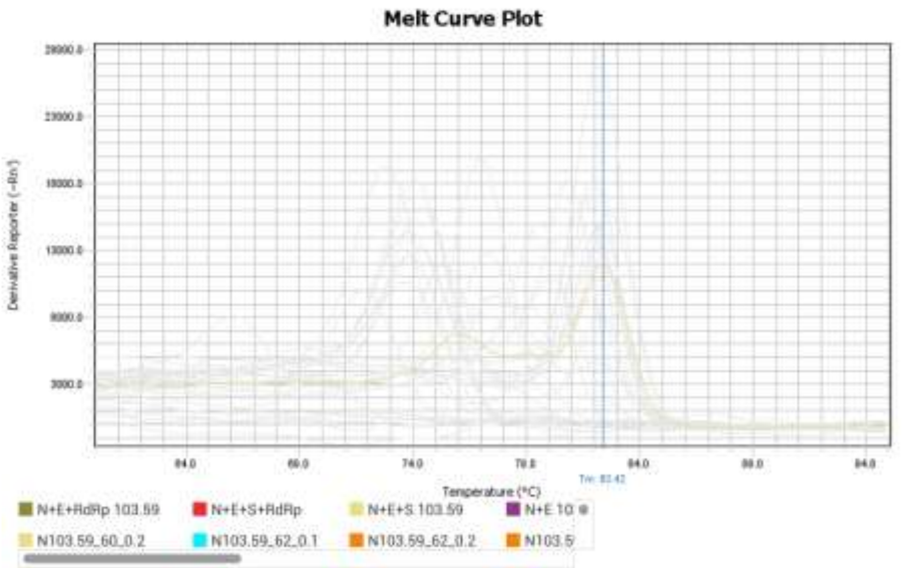

Melt curve plot of the triplex assay using JUST_N1+ JUST_E1+ JUST_S1 primer combination.

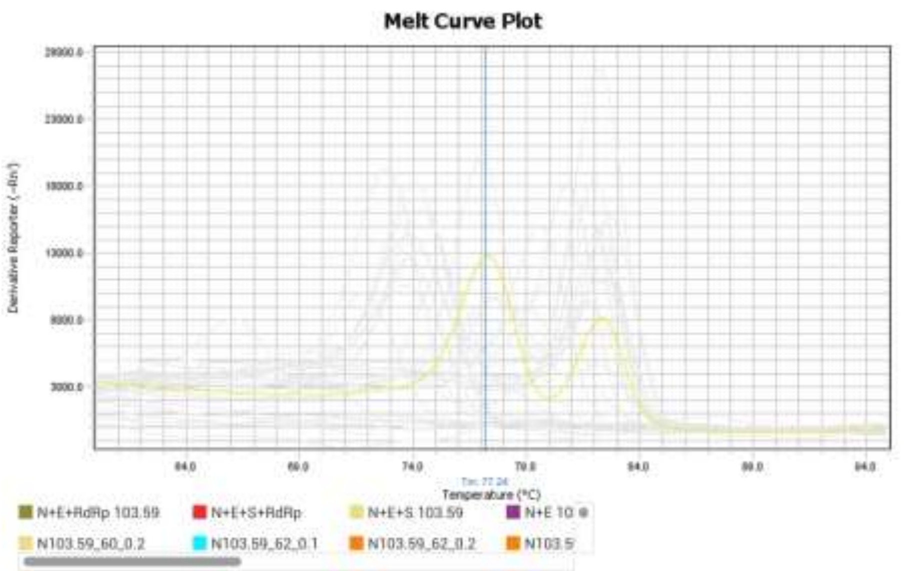	Melt curve plot of the triplex assay using JUST_N1+ JUST_RdRp1+ JUST_S1 primer combination.
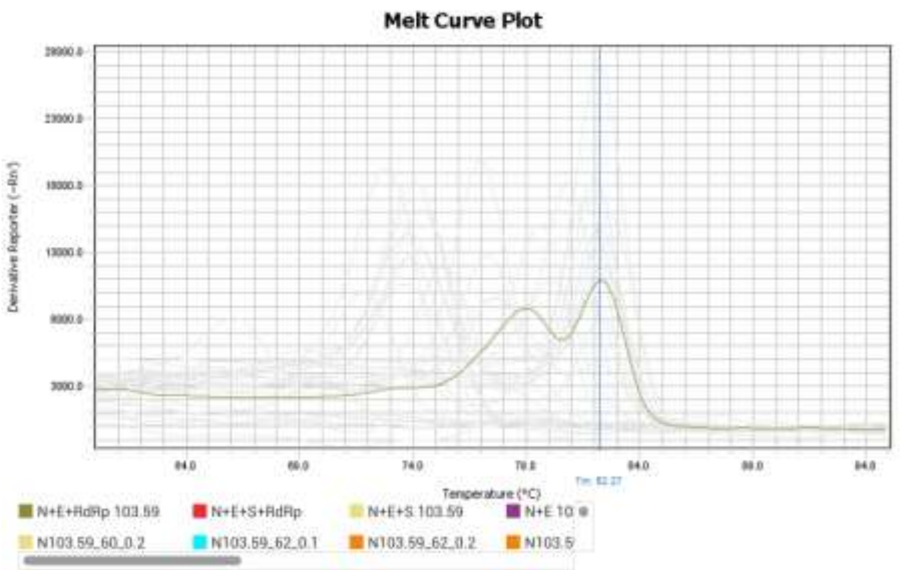	Melt curve plot of the triplex assay using JUST_N1+ JUST_RdRp1+ JUST_E1 primer combination.

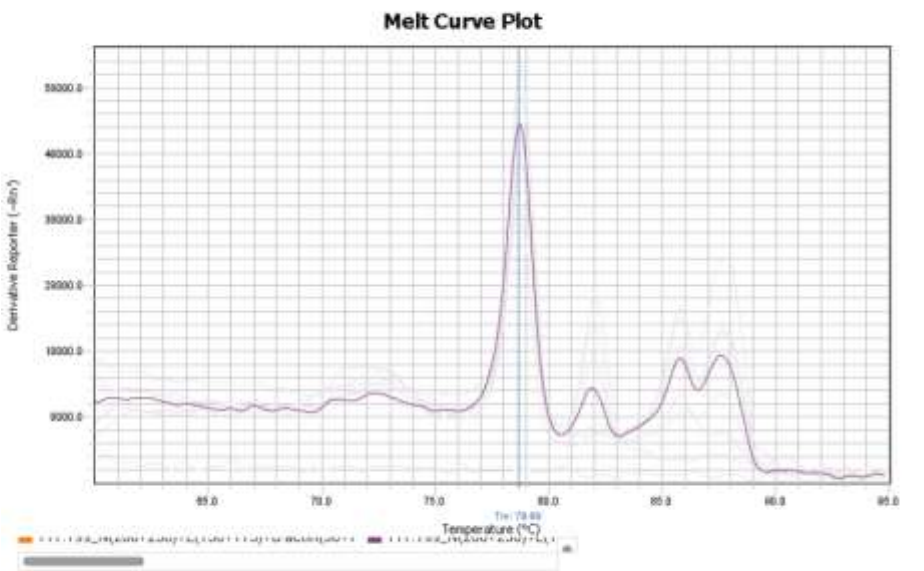	Melt curve plot of the triplex assay using JUST_N1+ JUST_E1+ Beta-actin primer combination.
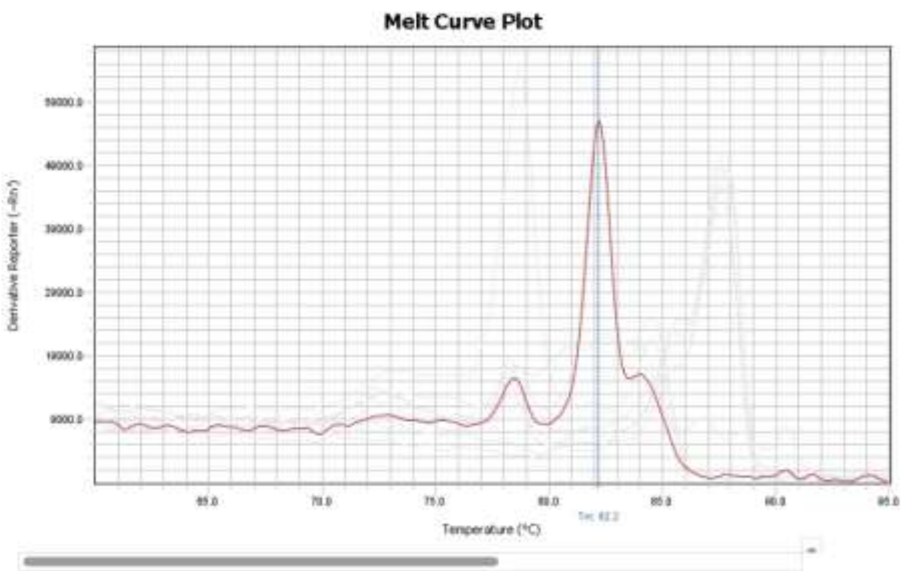	Melt curve plot of the triplex assay using JUST_N1+ JUST_E1+ GAPDH primer combination.

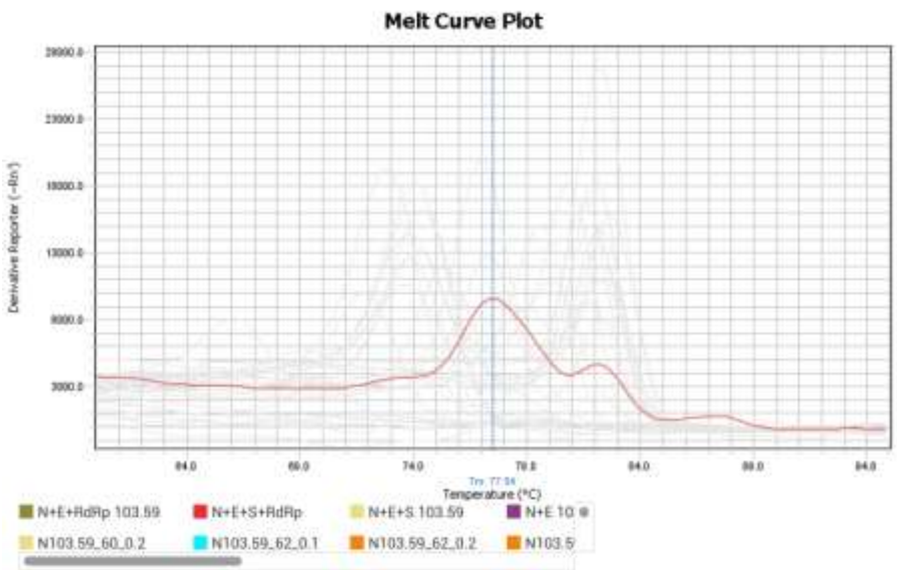	Melt curve plot of the quadruplex assay using JUST_N1+ JUST_E1+ JUST_S1+ JUST_RdRp1 primer combination.

Supplementary Fig. S4: Melting curve plot of selected genes in different primer conditions.

<table>
<tr>
<td>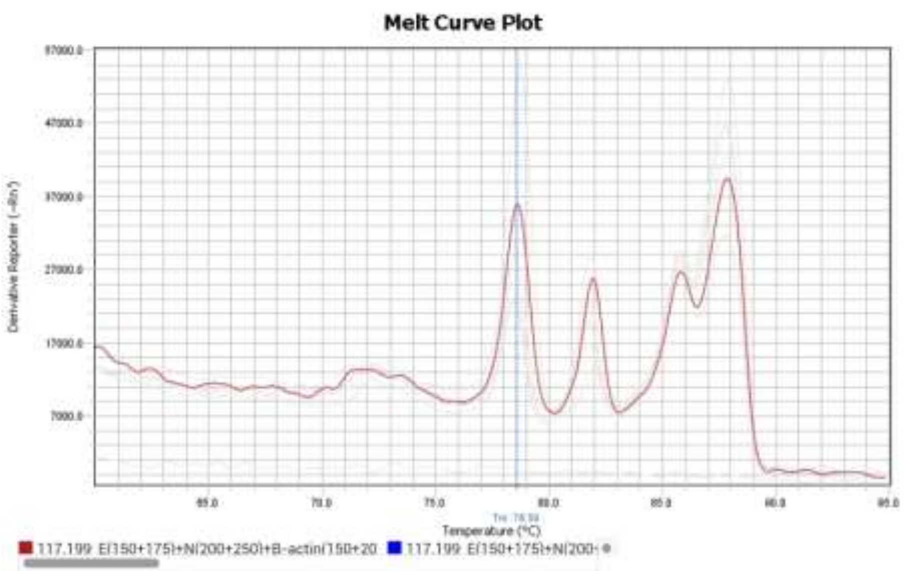
</td>
<td>Melt curve plot of the triplex assay using JUST_N1+ JUST_E1+ Beta-actin primer combination. Primer concentration as follow: JUST_N1_F=200 nM, JUST_N1_R=250 nM, JUST_E1_F=150 nM, JUST_E1_R=175nM, Beta-actin_F=150 nM and Beta-actin_R=200 nM</td>
</tr>
<tr>
<td>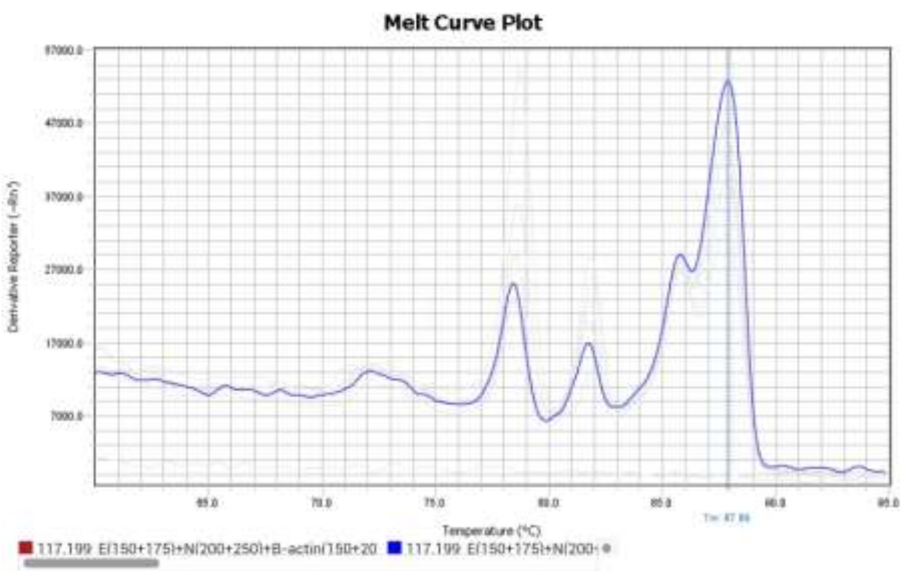
</td>
<td>Melt curve plot of the triplex assay using JUST_N1+ JUST_E1+ Beta-actin primer combination. Primer concentration as follow: JUST_N1_F=200 nM, JUST_N1_R=250 nM, JUST_E1_F=150 nM, JUST_E1_R=175nM, Beta-actin_F=200 nM and Beta-actin_R=250 nM</td>
</tr>
</table>

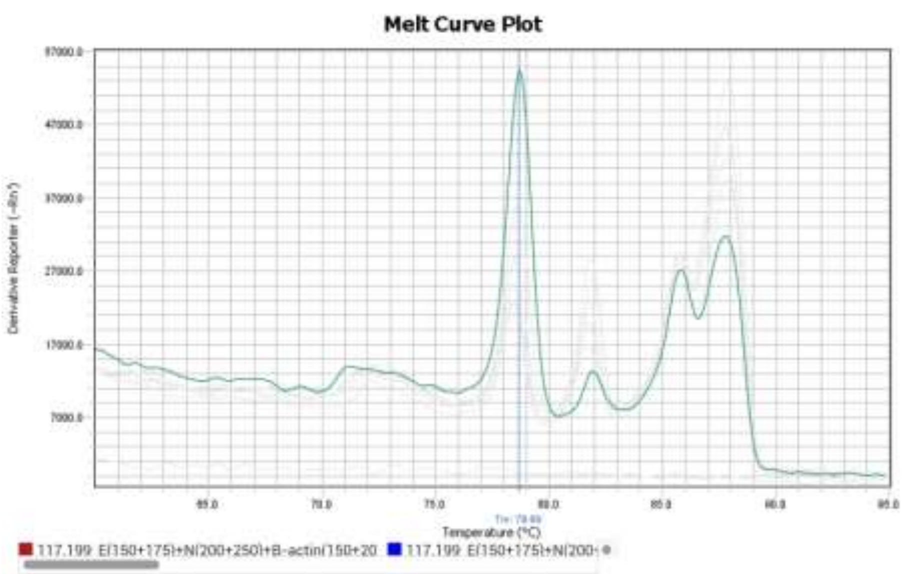	Melt curve plot of the triplex assay using JUST_N1+ JUST_E1+ Beta-actin primer combination. Primer concentration as follow: JUST_N1_F=300 nM, JUST_N1_R=350 nM, JUST_E1_F=150 nM, JUST_E1_R=175nM, Beta-actin_F=150 nM and Beta-actin_R=200 nM
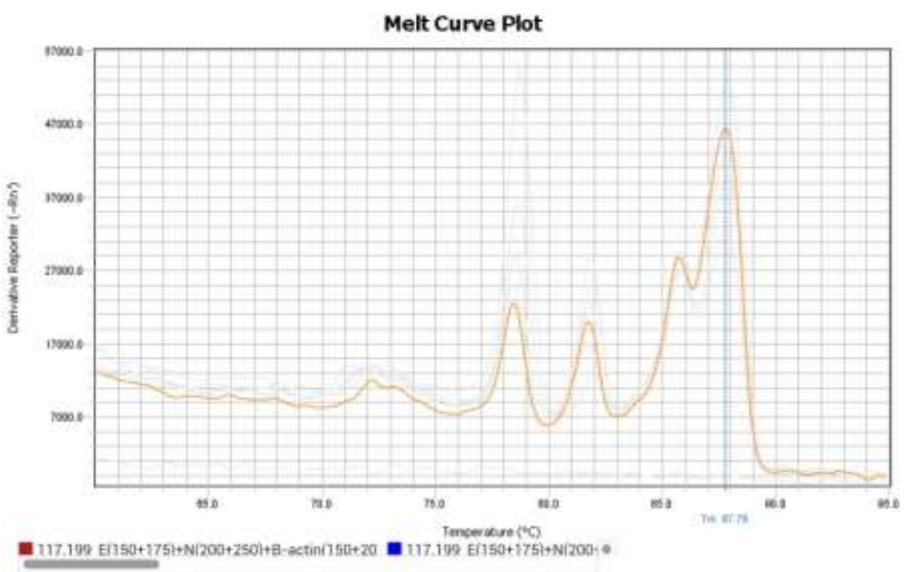	Melt curve plot of the triplex assay using JUST_N1+ JUST_E1+ Beta-actin primer combination. Primer concentration as follow: JUST_N1_F=300 nM, JUST_N1_R=350 nM, JUST_E1_F=150 nM, JUST_E1_R=175nM, Beta-actin_F=200 nM and Beta-actin_R=250 nM

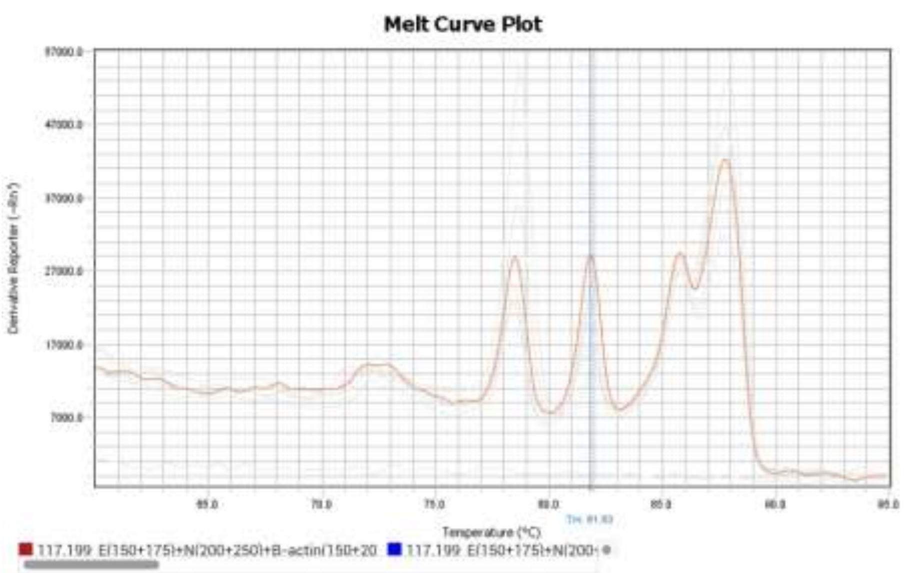	Melt curve plot of the triplex assay using JUST_N1+ JUST_E1+ Beta-actin primer combination. Primer concentration as follow: JUST_N1_F=300 nM, JUST_N1_R=400 nM, JUST_E1_F=150 nM, JUST_E1_R=175nM, Beta-actin_F=150 nM and Beta-actin_R=200 nM
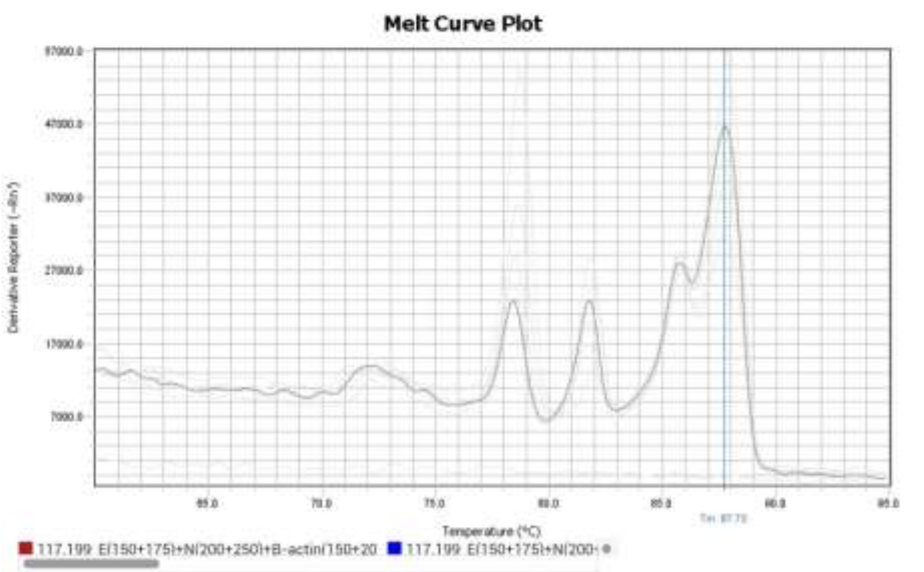	Melt curve plot of the triplex assay using JUST_N1+ JUST_E1+ Beta-actin primer combination. Primer concentration as follow: JUST_N1_F=300 nM, JUST_N1_R=400 nM, JUST_E1_F=150 nM, JUST_E1_R=175nM, Beta-actin_F=200 nM and Beta-actin_R=250 nM

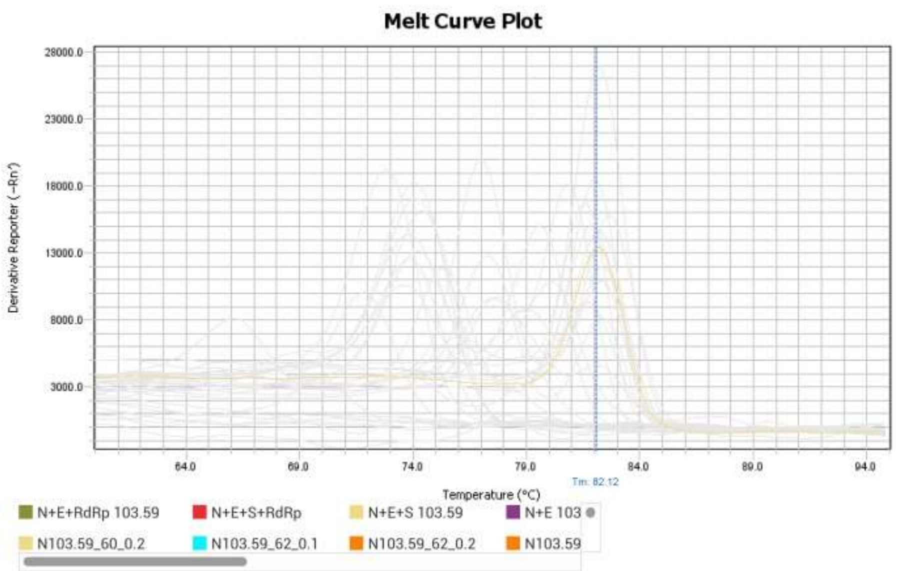	Melt curve plot for JUST_N1. Primer concentration 100 nM and Annealing Temperature 60°C
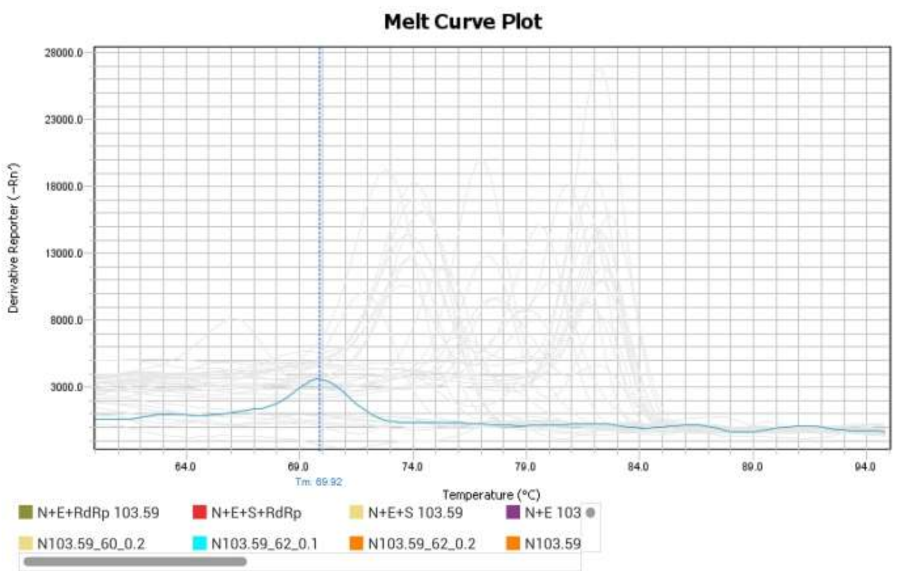	Melt curve plot for JUST_N1. Primer concentration 50 nM and Annealing Temperature 60°C

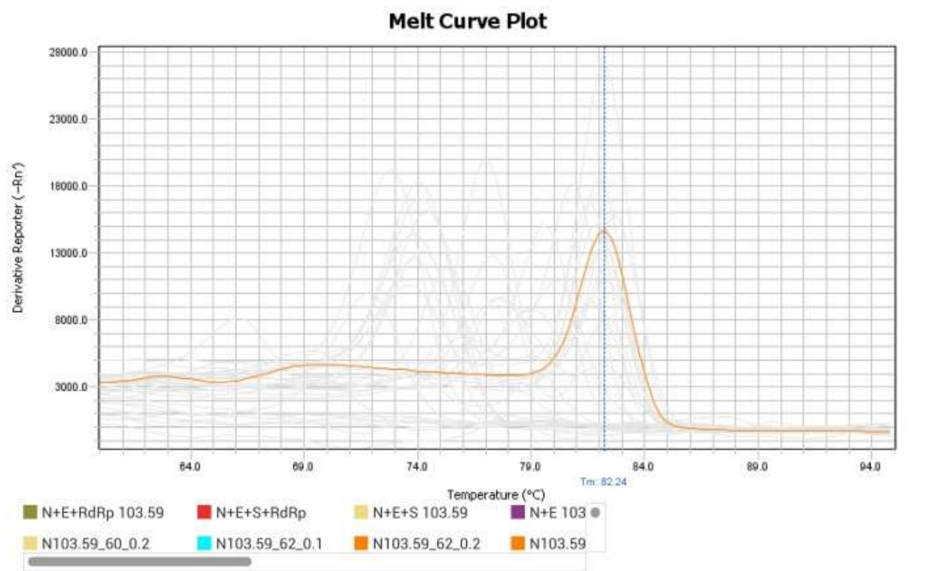	Melt curve plot for JUST_N1. Primer concentration 100 nM and Annealing Temperature 62℃
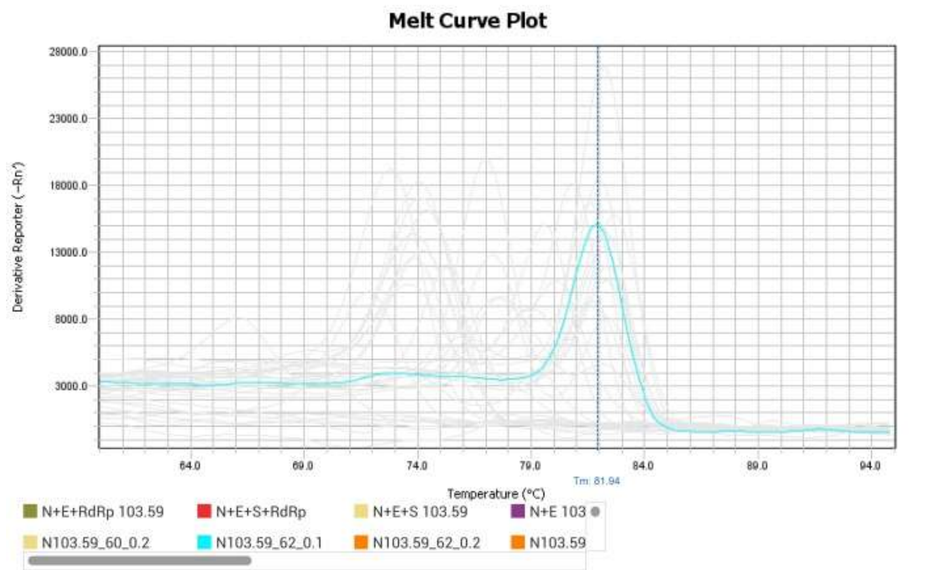	Melt curve plot for JUST_N1. Primer concentration 50 nM and Annealing Temperature 62℃

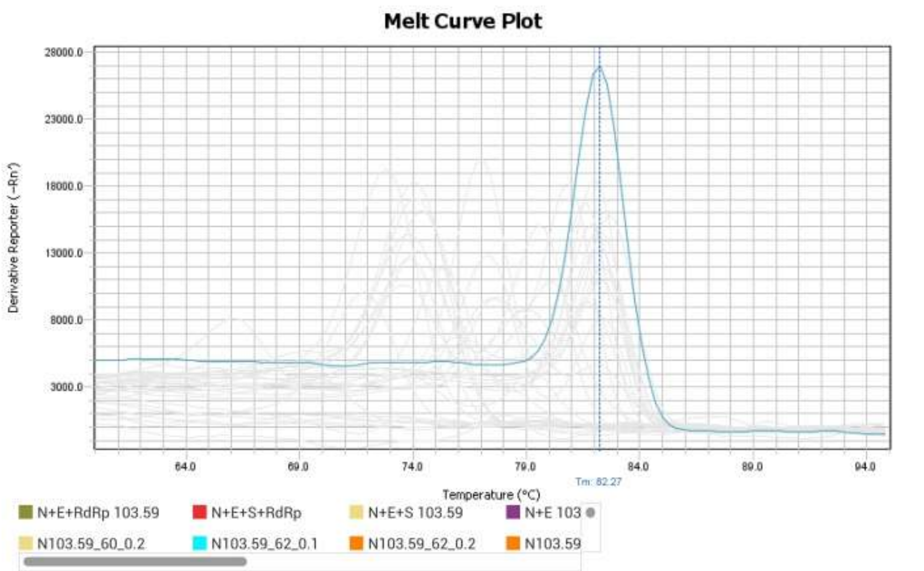

Melt curve plot for JUST_N1. Primer concentration 100 nM and Annealing Temperature 64℃

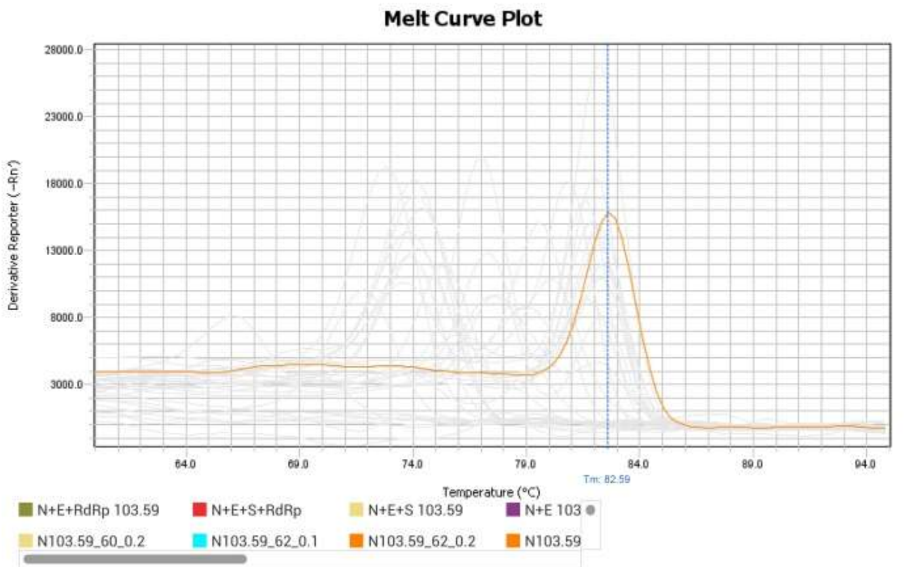

Melt curve plot for JUST_N1. Primer concentration 50 nM and Annealing Temperature 64℃

Supplementary Fig. S5: Gel Run image of the contradictory samples and representative concordant positive samples.

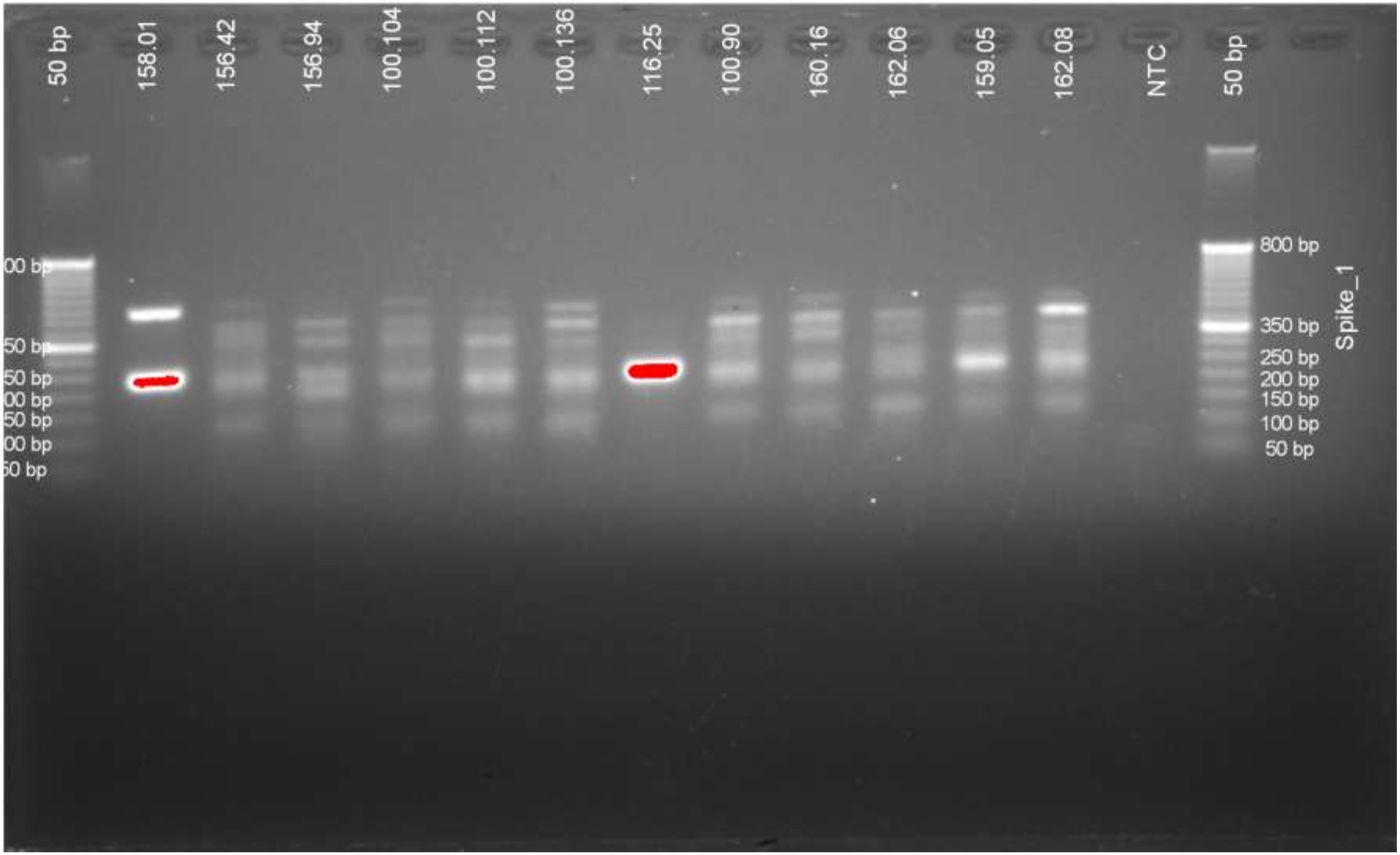

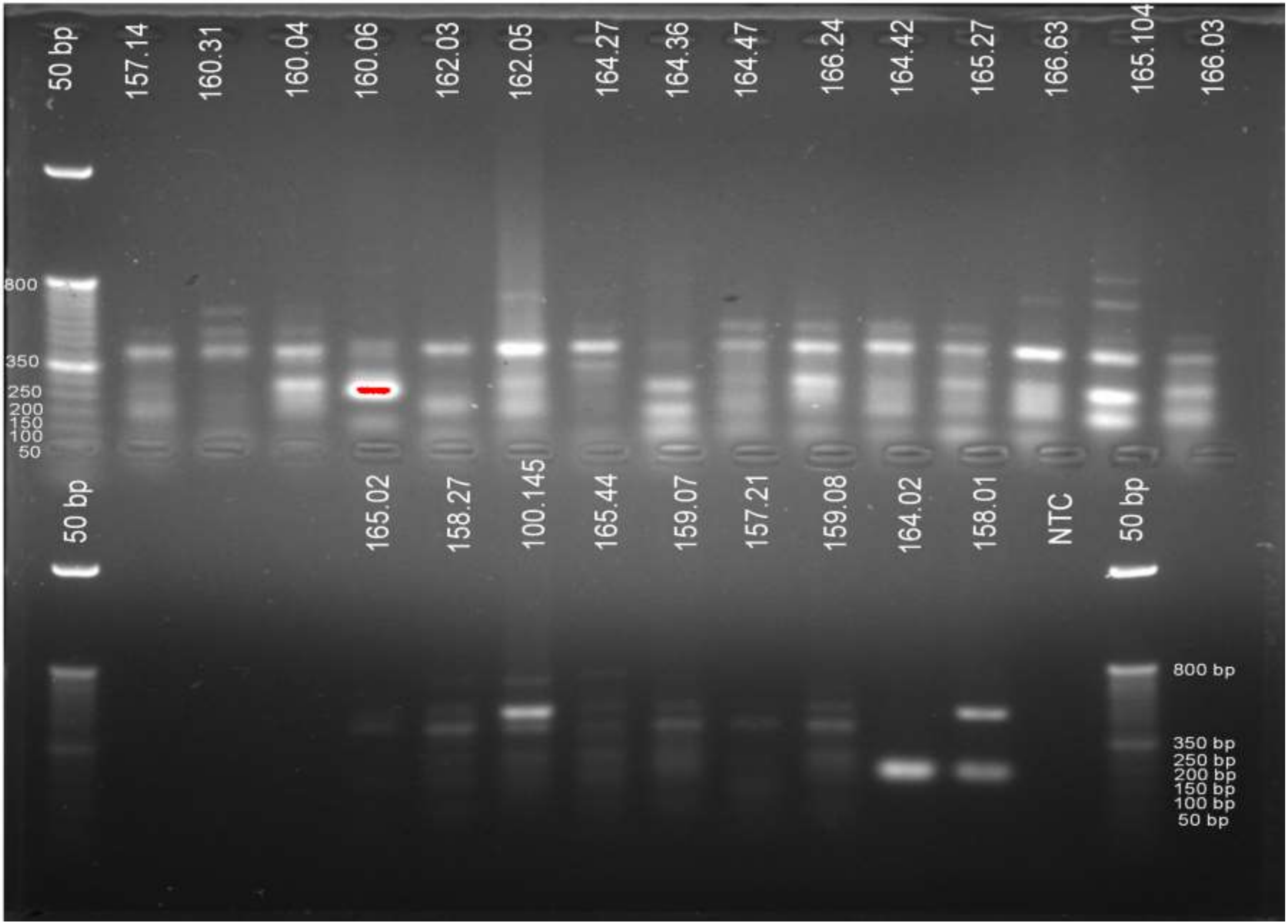

50 bp
157.14
160.31
160.04
160.06
162.03
162.05
164.27
164.36
164.47
166.24
164.42
165.27
166.63
165.104
166.03
800
350
250
200
150
100
50
50 bp
165.02
158.27
100.145
165.44
159.07
157.21
159.08
164.02
158.01
NTC
50 bp
800 bp
350 bp
250 bp
200 bp
150 bp
100 bp
50 bp

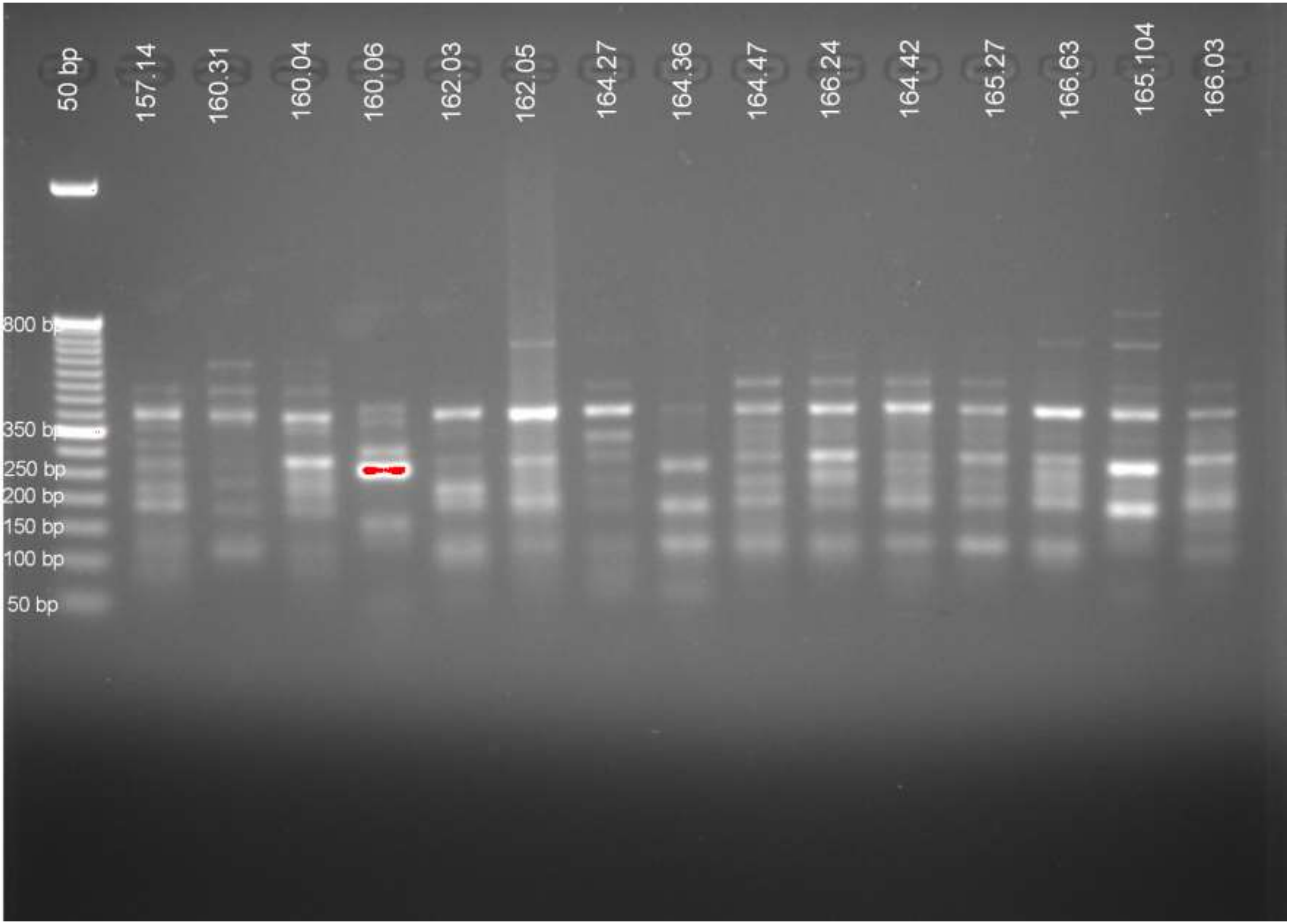

50 bp
157.14
160.31
160.04
160.06
162.03
162.05
164.27
164.36
164.47
166.24
164.42
165.27
166.63
165.104
166.03
800 bp
350 bp
250 bp
200 bp
150 bp
100 bp
50 bp

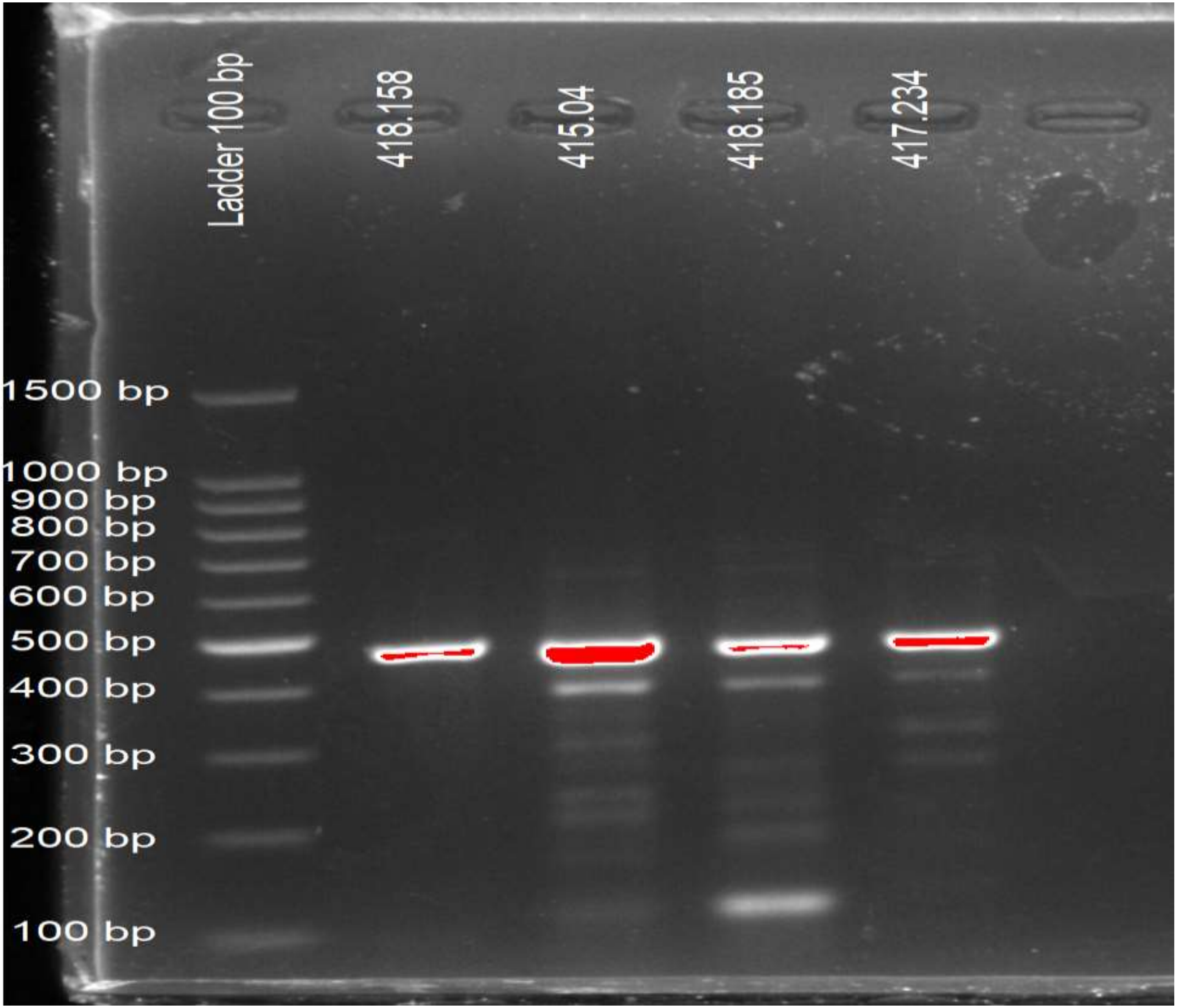
Ladder 100 bp
418.158
415.04
418.185
417.234
1500 bp
1000 bp
900 bp
800 bp
700 bp
600 bp
500 bp
400 bp
300 bp
200 bp
100 bp

Supplementary Fig. S6

Species/Ab	Group N	*	*	*	*	*	*	*	*	*	*	*	*	*		*	*	*	*	*	*	*
1. Refseq		C	T	C	A	A	G	T	G	T	C	T	G	T	G	G	A	T	C	A	C	G
2. 164.02		C	T	C	A	A	G	T	G	T	C	T	G	T	K	G	A	T	C	A	C	G
3. 164.36		C	T	C	A	A	G	T	G	T	C	T	G	T	G	G	A	T	C	A	C	G
4. 165.104		C	T	C	A	A	G	T	G	T	C	T	G	T	G	G	A	T	C	A	C	G
